# Supplementary material for: The structure of the native cardiac crossbridge in the rigor state
Source: Sci Adv. 2026 Aug 26;12(35):eaeg1209. doi: 10.1126/sciadv.aeg1209 (PMC13510625; doi:10.1126/sciadv.aeg1209)
Supplement: Supplementary file 1 [file sciadv.aeg1209_sm.pdf]

Science Advances
AAAS

Supplementary Materials for

The structure of the native cardiac crossbridge in the rigor state

Cristina M. Risi *et al.*

Corresponding author: Vitold E. Galkin, galkinve@odu.edu

Sci. Adv. **12**, eaeg1209 (2026)
DOI: 10.1126/sciadv.aeg1209

The PDF file includes:

Supplementary Materials and Methods
Figs. S1 to S20
Legend for movie S1
Tables S1 to S7
References

Other Supplementary Material for this manuscript includes the following:

Movie S1

MATERIALS AND METHODS

Imaging and image analysis. Details of imaging conditions and image reconstruction are provided in Table S1. Micrographs were collected on a 300-kV Titan Krios electron microscope equipped with a K3 direct electron detector and an energy filter operating in super-resolution mode. Micrographs were recorded in 40 subframes at a dose rate of ~0.85 e-/Å2 per frame over a defocus range of 0.5–3.5 µm, with a pixel size of 0.678 Å. All processing was performed in RELION (*47*). Images were imported into RELION for motion correction and dose weighting using internal RELION algorithms. The images were binned to a pixel size of 1.356 Å, and contrast transfer function (CTF) parameters were determined using CTFFIND4 (*77*). Particle selection was performed manually from 41,405 micrographs to extract 5,235,506 cTF segments (537X537 Å with 27.4 Å overlap) having HMM sparsely bound (fig. S4A, red arrows). Particles were decimated to 4.068 Å/px (figs. S4B and S5, A to B). To evaluate HMM interactions along the length of the entire regulatory unit (RU), which comprises seven actin molecules, we generated low-resolution models of cTF with an attached two myosin heads (fig. S5C, short surfaces) to cover all possible positions of HMM within the TF RU. 40 classes based on the relative position of the Tn complex (fig. S5C, red arrows) and HMM (fig. S5C, green arrows) were generated. The resulting class averages differed from the initial models due to the presence of RLCs in the myosin neck region (fig. S5C, long surfaces). Because cTF is comprised of two strands, some classes were redundant in terms of HMM binding to cTF RU. We used segments from the following five classes (fig. S5C): (1) Class39 from the 40-class sorting, representing HMM bound to actins 6 and 7 of the RU (referred to as Class 6-7); (2) Class31 with HMM bound to actins 5 and 6 (referred to as Class 5-6); (3) Class11 with HMM bound to actins 2 and 3 (referred to as Class 2-3); (4) Class10 with HMM bound to actins 1 and 2 (referred to as Class 2-3); (5) Class37 representing HMM

bound to actins 7 and 1 (referred to as Class 2-3) for 3D focused refinement (*41-43*). Details for the refinement of each class are provided below.

Class 6-7: The overview of the Class 6-7 reconstruction is shown in fig. S7. The class output from the initial position sorting (fig. S7A, hot pink surface), in addition to the expected position of HMM relative to the Tn core (fig. S7A, blue arrow), contained traces of additional HMM molecules bound elsewhere (fig. S7A, magenta and brown arrows), suggesting heterogeneity within the class. To overcome this, three individual templates were built using the initial class average (fig. S7A, hot-pink surface) as a guide. Templates 1 (fig. S7B, orange surface) and 2 (fig. S7B, purple surface) contained additional HMM molecules above or below the expected position (fig. S7B, brown and blue arrows or magenta and blue arrows, respectively), while the third template had only one HMM molecule (fig. S7B, green surface and blue arrow). Templates were low-pass filtered to 15 Å and used for supervised 3D classification in RELION (*47*) (fig. S7B, solid surfaces). As expected, double-decorated classes represented a small fraction of the class: double HMM occupancy mode 1 (n=11,675) (fig. S7C, orange surface), double HMM occupancy mode 2 (n=10,791) (fig. S7C, purple surface), and single HMM occupancy (n=86,644) (fig. S7C, green surface). The class representing the single HMM occupancy (n=86,644) (fig. S7C, red box) was re-extracted at the full scale of 1.356 Å/px, 3D-refined starting from the class average low-pass filtered to 15 Å (fig. S7C, green surface), and post-processed (fig. S7D). To obtain a high-resolution map, the particle polishing algorithm in RELION was used for reference-based beam-induced motion correction. The regulatory light chains (RLCs) were not well resolved in the initial refinement (fig. S7D, black arrow), suggesting that, due to disorder, RLCs required further classification (discussed in detail later). Finally, to obtain a high-resolution structure of the HMM bound to actin molecules 6 and 7, a focused refinement was done using a 10 Å mask that excluded

the opposite TF strand, distal actin molecules, and RLCs (fig. S7E, red surface). The resulting high-resolution density map was segmented (fig. S7F, yellow surface) and used to model (fig. S7G, cyan ribbons).

The global resolution for the map (4.3 Å) (fig. S8A) was calculated in RELION using the gold-standard FSC criterion of 0.143, which measures the normalized cross-correlation between the two half-maps in Fourier space. To do this, the entire particle subset is randomly split into two halves, and each half is independently reconstructed into a density map. This method of resolution determination is problematic when a cryo-EM density map exhibits resolution variability across local regions due to heterogeneity or intrinsic disorder, and it also yields the most pessimistic resolution due to its bias to the set size (*46, 48, 78*). We used RELION to calculate a local resolution map, which showed a range of 3.8-9.2 Å (fig. S8B). The region resolved at the highest resolution of 3.8 Å included actin and the actomyosin interface (fig. S8B, black arrows), whereas the ELC interface with the myosin motor domain was resolved at 4.2 Å (fig. S8B, black arrow). As expected, the distal portions of the map were the lowest resolution (fig. S8B, red arrows). Given the drawbacks of the RELION resolution-determination algorithm discussed above, we also used CryoRes, a deep-learning-based algorithm (*48*), to determine local resolution from the cryo-EM density map (fig. S8C). The resolution estimate ranged from 3.79 Å to 4.27 Å within the map (fig. S8C), consistent with the RELION local resolution determination. That resolution range was supported by the map's quality, enabling visualization of side chains in the cryo-EM map (fig. S8D). Detailed views of the map components (actin, myosin motor domain, myosin CM-loop, loop-2, loop-3, loop-4, HLH-motif, and the ELC interface) at 3.8-4.2 Å resolution that are discussed in the paper are shown along with the atomic model to illustrate the quality of the data (fig. S8D).

Class 5-6: The overview of the Class 5-6 reconstruction is shown in fig. S9. The class output from the initial position sorting (fig. S9A, hot pink surface) contained traces of HMM near the lower region of the filament (fig. S9A, purple arrow). Two templates were generated using the initial class average (fig. S9A, hot-pink surface) as a reference. Template 1 (fig. S9B, yellow surface) contained an HMM molecule at its expected position (fig. S9B, orange arrow) while the second template (fig. S9B, cyan surface) contained an additional HMM molecule where the traces were observed (fig. S9B, purple and orange arrows). The templates were low-pass filtered to 15 Å and used for supervised 3D classification in RELION (fig. S9B, solid surfaces). Consistent with the initial class average, the majority of segments were classified as having one HMM molecule bound to actins 5 and 6 (n=55,229) (fig. S9C, yellow surface), with a few segments possessing two HMM molecules bound (n=5,451) (fig. S9C, cyan surface). The primary class (fig. S9C, red box) was re-extracted at 1.356 Å/px and reconstructed (fig. S9D) starting from the class average (fig. S9C, yellow surface), low-pass filtered to 15 Å. To obtain a high-resolution map, the particle polishing algorithm was applied before focused refinement. To get the high-resolution structure, we used a 10 Å mask that excluded the opposing TF stand and distal actin molecules, as well as the RLCs (fig. S9E, green surface). The high-resolution density map was segmented (fig. S9F, lavender surface) and used for modeling (fig. S9G, blue ribbons).

The global (4.6 Å) (fig. S10A) and local resolutions (4.0 – 8.6 Å) (fig. S10B) were calculated using RELION's built-in algorithms. A local resolution map generated using RELION yielded the highest resolution of 4.0 Å (fig. S10B, black arrows) within actin, the myosin motor domain, and at the actomyosin interface. The resolution estimation at the ELC interface was 4.4 Å (fig. S10B, black arrow). The resolution estimation determined by CryoRes (*48*) ranged from 3.85 Å to 4.3 Å (fig. S10C), which was in line with the RELION local resolution map. Resolution

estimates were consistent with the details in the cryo-EM map (fig. S10D). Detailed views of the components of the map, along with associated atomic models discussed in the manuscript, are depicted in fig. S10D.

Class 2-3: Details of the processing are shown in fig. S11. The class average from the initial position sorting (fig. S11A, hot pink surface) contained traces of additional HMM molecules bound to actins rather than 2 and 3 (fig. S11A, dark red, chartreuse, purple, brown, teal, and orange arrows). To sort out those minor modes, four templates were made using the initial class average (fig. S11, hot pink surface) as a reference. Template 1 (fig. S11B, yellow surface) contained one HMM molecule bound to actins 2 and 3 (fig. S11B, orange arrow), template 2 contained three HMM molecules (fig. S11B, green surface, orange, brown, and teal arrows), template 3 (fig. S11B, lavender surface) also contained three HMM molecules (fig. S11B, orange, dark red, and chartreuse arrows), and template 4 (fig. S11B, cyan surface) contained two HMM molecules (fig. S11B, orange and purple arrows). The templates were low-pass filtered to 15 Å and used for supervised 3D classification in RELION (fig. S11B, solid surfaces). The frequencies of resultant modes are as follows: single HMM occupancy (n=63,599) (fig. S11C, yellow surface), triple HMM occupancy mode 1 (n=2,783) (fig. S11C, green surface), triple HMM occupancy mode 2 (n=2,916) (fig. S11C, lavender surface), and double HMM occupancy (n=10,691) (fig. S11C, cyan surface). The main class with HMM bound to actins 2 and 3 (n=63,599) (fig. S11C, yellow surface and brown arrow) had weaker-than-expected density for the Tn core (fig. S11C, yellow surface and grey arrow), suggesting that HMM bound next to the Tn core might cause partial dissociation of the Tn complex. To obtain the structure of the intact cross-bridge, segments selected in (C) were sorted by the integrity of the Tn tandem (fig. S11D, solid surfaces). The frequencies of resultant modes are as follows: intact Tn tandem (n=31,180) (fig. S11E, magenta surface) and upper Tn

occupancy (n=32,419) (fig. S11E, blue surface). The class with the intact Tn tandem (fig. S11E, magenta surface, red box) was re-extracted, refined, and post-processed at 1.356 Å/px (fig. S11F), starting from the class average, low-pass filtered to 15 Å (fig. S11E, magenta surface). To obtain a high-resolution map, the particle polishing algorithm was applied before focused refinement. Next, we used a focused refinement approach (fig. S11) by utilizing a 10 Å mask that excluded the opposite strand of TF, along with distal actins and RLCs, from the refinement process (fig. S11E, red surface). The high-resolution density map was segmented (fig. S11F, green surface) and used for modeling (fig. S11I, purple ribbons).

The global (4.8 Å) (fig. S12A) and local resolutions (4.2 – 8.7 Å) (fig. S12B) were calculated using RELION. The local resolution map yielded the highest resolution of 4.2 Å (fig. S12B, black arrows) within actin, myosin motor domain, and at the actomyosin interface (fig. S12B). CryoRes (*48*) resolution estimate ranged from 4.27 to 4.68 Å (fig. S12C). This local resolution determination was consistent with visualization of large amino acid side chains in the cryo-EM map (fig. S12D).

<u>Class 1-2</u>: Details are shown in fig. S13. The class average from the initial position sorting (fig. S13A, hot pink surface) contained traces of extra density at the top and bottom of the map (fig. S13A, light grey, cornflower, light green, gold, dark red, dark grey, teal, light purple, sienna, deep pink, and khaki arrows), suggesting heterogeneity. Twelve templates were made based on the initial class average (fig. S13A, hot pink surface). The first template (fig. S13B, cerulean surface) contained an HMM molecule bound to actins 1 and 2 (fig. S13B, sienna arrow). In addition we generated ten templates possessing two or three HMM molecules bound to the TF: template 1 (fig. S13B, chartreuse surface, sienna and dark red arrows), 2 (fig. S13B, lavender surface, sienna and dark grey arrows), 3 (fig. S13B, magenta surface, sienna and khaki arrows), 4 (fig. S13B, orange

surface, sienna and deep pink arrows), 5 (fig. S13, green surface, sienna and teal arrows), 6 (fig. S13B, red surface, sienna and light green arrows), 7 (fig. S13B, purple surface, sienna and cornflower arrows), 8 (fig. S13B, yellow surface, sienna and light grey arrows), 9 (fig. S13B, blue surface, sienna and light purple arrows), and template 10 (fig. S13B, dark green surface, sienna and gold arrows). There was one triple HMM occupancy mode (fig. S13B, cyan surface, teal, sienna, and dark green arrows). The templates were low-pass filtered to 15 Å and used for supervised 3D sorting in RELION (fig. S13B, solid surfaces). The frequencies of resultant modes were as follows: single HMM occupancy (n=54,451) (fig. S13C, cerulean surface), double HMM occupancy mode 1 (n=508) (fig. S13C, chartreuse surface), double HMM occupancy mode 2 (n=267) (fig. S13C, lavender surface), double HMM occupancy mode 3 (n=438) (fig. S13C, magenta surface), double HMM occupancy mode 4 (n=289) (fig. S13C, orange surface), double HMM occupancy mode 5 (n=4,391) (fig. S13C, green surface), double HMM occupancy mode 6 (n=5,249) (fig. S13C, red surface), double HMM occupancy mode 7 (n=1,670) (fig. S13C, purple surface), double HMM occupancy mode 8 (n=151) (fig. S13C, yellow surface), double HMM occupancy mode 9 (n=5,447) (fig. S13C, blue surface), double HMM occupancy mode 10 (n=2,210) (fig. S13C, dark green surface), and triple HMM occupancy (n=120) (fig. S13C, cyan surface). The primary class representing one HMM molecule bound to actins 1 and 2 (n=54,451) (fig. S13C, cerulean surface, red box) was re-extracted, refined, and post-processed at a raster of 1.356 Å/px (fig. S13D) starting from the class average filtered to 15 Å (fig. S13C, cerulean surface). To achieve a high-resolution map, the particle polishing algorithm was used for reference-based beam-induced motion correction (fig. S13D). Next, we used focused refinement to obtain the high-resolution 3D reconstruction (fig. S13, E to G). This was done by utilizing a 10 Å mask that excluded the TF opposite strand, distal actins, and RLCs (fig. S13E, light yellow

surface). The resultant density map was segmented (fig. S13F, light pink surface) and used for modeling (fig. S13G, dark brown ribbons).

The global (4.5 Å) (fig. S14A) and local resolutions (3.9–8.5 Å) (fig. S14B) were calculated using RELION's built-in algorithms. The local resolution map calculated by RELION was consistent with resolution estimation from CryoRes (*48*) and ranged from 3.78 to 4.2 Å within actin, myosin motor domain, and ELCs interface with myosin (fig. S14, B and C). Close-up views of the map and the atomic model in the regions of interest discussed in the manuscript are shown to illustrate the quality of the data (fig. S14D).

<u>Class 7-1</u>: Details are shown in fig. S15. The class average from the initial position sorting (fig. S15A, hot pink surface) contained traces of extra density along the entire density map (fig. S15A, dark grey, cornflower, light green, deep pink, peach, light grey, sienna, dark brown, teal, and khaki arrows), indicating pronounced heterogeneity within the class. To characterize it, 11 templates were generated using the initial class average (fig. S15A, hot-pink surface) as a guide. Template 1 (fig. S15B, cyan surface) contained one HMM molecule bound to actins 7 and 1 (fig. S15B, sienna arrow). In addition we generated nine templates for multiple HMMs bound to TF: template 1 (fig. S15B, blue surface, sienna and light green arrows), template 2 (fig. S15B, purple surface, sienna and deep pink arrows), 3 (fig. S15B, orange surface, sienna and khaki arrows), 4 (fig. S15B, dark green surface, sienna and dark brown arrows), template 5 (fig. S15B, yellow surface, sienna and teal arrows), 6 (fig. S15B, red surface, sienna and dark grey arrows), 7 (fig. S15B, green surface, sienna and peach arrows), 8 (fig. S15B, lavender surface, sienna and light grey arrows), and template 9 (fig. S15B, magenta surface, sienna and cornflower arrows). There was one triple HMM occupancy mode (fig. S15B, gold surface, light green, sienna, and dark grey arrows). The templates were low-pass filtered to 15 Å and used for supervised 3D sorting in RELION (fig.

S15B, solid surfaces). The frequencies of resultant modes were as follows: single HMM occupancy (n=66,037) (fig. S15C, cyan surface), double HMM occupancy mode 1 (n=988) (fig. S15C, blue surface), double HMM occupancy mode 2 (n=1,248) (fig. S15C, purple surface), double HMM occupancy mode 3 (n=237) (fig. S15C, orange surface), double HMM occupancy mode 4 (n=1,460) (fig. S15C, dark green surface), double HMM occupancy mode 5 (n=509) (fig. S15C, yellow surface), double HMM occupancy mode 6 (n=1,537) (fig. S15C, red surface), double HMM occupancy mode 7 (n=507) (fig. S15C, green surface), double HMM occupancy mode 8 (n=1,347) (fig. S15C, lavender surface), double HMM occupancy mode 9 (n=1,089) (fig. S15C, magenta surface), and the triple HMM occupancy (n=56) (fig. S15C, gold surface). The class with a single HMM molecule (fig. S15C, cyan surface, red box) had two junction regions (fig. S15C, black asterisks), suggesting the set was a mixture of particles having HMM bound to either actin molecules 6-7 or actin molecules 7-1. Segments were sorted by the position of HMM (fig. S15D) to select particles with the HMM bound to actin molecules 7-1 (fig. S15D, cerulean surfaces). The sorting indicated that the class predominantly was comprised of segments having HMM bound to actins 6 and 7: HMM bound to actin 7-1 (n=19,798) (fig. S15E, cerulean surface) and HMM bound to actin molecules 6-7 (n=46,239) (fig. S15E, chartreuse surface). Due to the small size of the Class 7-1 set, we decided not to pursue the refinement of this class.

Statistical analysis of class frequencies: *Statistical analysis of overall classes frequences:* Class frequencies for each structural class (1-2, 2-3, 5-6, 6-7, and 7-1; Fig. 1) were independently quantified for the three datasets from independent thin-filament preparations (grids 1-3, 4-6, and 7-10) as described above, ensuring that only HMM-decorated filaments were included in the analysis; naked actin and naked cTF particles were excluded. The grids 1-3 set contained 634,205 HMM-decorated particles; the grids 4-6 set contained 1,113,851 particles; and the grids 7-10 set

contained 1,391,003 particles. Because the total particle numbers differed among the three independent experiments, the raw counts were converted to relative percentages. The frequencies for each class are shown in Table S3. For each HMM class (1-2, 2-3, 5-6, 6-7, and 7-1), the average frequency (Mean) and standard deviation (SD) were calculated across the three sets using GraphPad Prism for Windows (version 11.0.2; Dotmatics, Boston, MA, USA) and are shown in Table S3. To determine if the mean myosin binding frequencies on the thin filament differed among the five distinct binding positions (Class 1–2, Class 2–3, Class 5–6, Class 6–7, and Class 7–1), a one-way analysis of variance (ANOVA) was performed (*79*). Replicate independence was maintained by using three completely independent sets of experimental data (N = 3 per group; N_{total} = 15). A one-way ANOVA F-statistic of 867.30 calculated in GraphPad Prism revealed a statistically significant difference ($p<0.001$ using a significance level of 0.05). An exploratory post hoc multiple-comparison profile was mapped using Tukey's honestly significant difference (HSD) test (*80*). Pairwise differences between group means and associated p-values were calculated in GraphPad Prism. The pairwise comparison data (Table S4) demonstrate that the frequencies of all five structural classes differ significantly from one another.

Statistical analysis of class frequencies on the two strands: Class frequencies for the five structural classes on both upper and lower strands of the thin filament are shown in Table S5. A two-way ANOVA: (a) confirmed a statistically significant difference among all 5 myosin-binding positions on the two strands ($F = 1336$ and $p<0.001$ using a significance level of 0.05); (b) indicated that strand on its own was not a significant factor ($p\sim1$); and (c) demonstrated a significant interaction between the class frequencies on the two strands ($F= 17.57$, $p<0.001$ using a significance level of 0.05). Myosin-binding class explained 98.3% of the total variance and the interaction term between class and strand explained the remainder. Next, an exploratory post hoc multiple-comparison

profile was mapped using Holm-Šídák's test (*81*) since it allowed specific comparisons (e.g., Class1-2 upper with Class1-2 lower, Class2-3 upper with Class2-3 lower, etc.). The pairwise comparison data (Table S6) demonstrate that the frequencies of Classes 1-2 and 2-3 are not statistically different between the two strands, whereas the differences in counts for Classes 5-6, 6-7, and 7-1 on the two strands are small but differ significantly from one another. To delve further into this unexpected, statistically identified interaction, we applied a χ^2 test in R (version 4.5.3) to assess whether the number of observations across the 5 myosin-binding classes on the upper strand is consistent with the observed average distribution on the lower strand (combining data from all grids). The results of this analysis ($\chi^2 = 14188$, df = 4, and $p < 2.2e^{-16}$) indicate that the number of observations of the 5 myosin-binding classes on the upper strand, with a distribution that appears very similar to that on the lower strand (Fig. S6A), is statistically inconsistent with that on the lower strand. We also performed the reverse analysis (number of observations in the 5 myosin-binding classes on the lower strand compared with the observed distribution on the upper strand, again using combined data from all grids), yielding similar results ($\chi^2 = 12248$, df = 4, and $p < 2.2e^{-16)}$. While the statistical effects related to the Tm strand are relatively small, their significance supports prior observations that the upper and lower strands of the cTF are not equivalent.

Modeling: Actin, Tm, HMM, and TnT1 were modeled separately before the refinement of the overall model. Modeling was performed similarly for all classes (e.g., Classes 6-7, 5-6, 2-3, and 1-2).

Actin modeling: To generate an atomic model of actin, four actin subunits were independently segmented from the focused refined map using the UCSF Chimera (*73*) segmenting tool (*82*). The segmented maps were auto-sharpened using the built-in algorithm in PHENIX (*76*). Actin subunits

from the porcine cardiac actomyosin complex (PDB 7JH7) (*12*) were rigid-body docked into the sharpened maps using UCSF Chimera (*73*). The four N-terminal residues were added to the models in UCSF Chimera (*73*) using the amino acid sequence (UniProt ID: B6VNT8). The atomic models were placed in ChimeraX (*83*-*84*) and flexibly fit with the ISOLDE algorithm (*75*). The models were then refined in PHENIX (*76*) using the real-space refinement algorithm to restore stereochemistry.

Tropomyosin and TnT1 modeling: To generate an initial structure of the cardiac porcine α-tropomyosin, we used its amino acid sequence (UniProt: P42639) and human α-tropomyosin from PDB 7KO5 (*41*) as a template. Human and porcine sequences are 98.59% identical (fig. S2B). The Tm junction region structure in the M-state was modeled using its structure in the Ca^{2+}-free state (PDB 8DD0 (*39*)) as a starting point. The initial model of Tm was rigid-body docked into the segmented Tm portion of the map using UCSF Chimera (*73*) and subsequently refined in Namdinator (*74*).

TnT1 helix was modeled using the AlphaFold database (AF-A0A5G2Q8N0-F1-v6) (*85, 86*). The TnT1 helix portion of the map was segmented, and the initial TnT1 model was docked into the map using UCSF Chimera (*73*), followed by refinement in PHENIX (*76*).

HMM modeling: To generate an initial model of the cardiac myosin using homology modeling in SWISS MODEL (*87*), we used the porcine cardiac amino acid sequence (UniProt: P79293) and the structure of skeletal myosin (PDB 5H53) (*15*) as a template. The two sequences shared 77.65 % identity. The structure 5H53 was selected as a template because it included a larger portion of the rod as well as loop 2, which was missing in the cardiac actomyosin structure 7JH7 (*12*). The ELC from the crystal structure PDB 6FSA (*50*) was used as a template for the porcine ventricular

amino acid sequence (UniProt: F1SNW4) in SWISS MODEL (*87*). The two amino acid sequences were 94.90% identical. The porcine cardiac myosin homology model was aligned to the motor domain in PDB 5H53 (*15*). The porcine cardiac ventricular isoform homology models for the ELCs and RLCs were also aligned to their structural counterparts in 5H53 (*15*). The components were combined into a single-headed myosin molecule and rigid-body docked into either the trailing or the leading heads, which were independently segmented from the overall map using the UCSF Chimera (*73*) segmenting tool (*82*). The initial refinement was performed using Namdinator (*74*) to correct for the significant movement of the tail region into the experimental density maps. Next, the models were iteratively refined in ChimeraX using the ISOLDE plugin (*75*) followed by real-space refinement in PHENIX (*76*).

Tn core modeling: We used the previously published model of the Ca^{2+}-bound fully activated state from the upper strand (PDB 8UZX) (*43*) as a template for the Tn core. The cardiac TnI homology model, with 10 additional N-terminal residues (i.e., residues 32-42), was generated using SWISS MODEL (*87*) and structurally aligned to the overall Tn core structure (PDB 8UZX (*43*)). Next, the initial Tn core structure was rigid-body docked into the segmented map using UCSF Chimera (*73*), followed by real-space refinement in PHENIX (*76*).

All the components (i.e., actin, Tm junction, TnT1 helix, myosin, Tn core, and ELC) were combined into a single model for each class. The parameters for the resultant models for each class are shown in Table S2.

Visualization of the RLCs of the HMM molecule bound to actin molecules 6 and 7: The polished consensus map was generated before the focused classification of the HMM RLC region (figs. S7D and S18A, grey surfaces). The HMM bound to actins 6 and 7, along with the portion of the

Tm junction and TnT1 helix (fig. S18A, cerulean surface), was segmented from the consensus map using the UCSF Chimera (*73*) segmenting tool (*82*) and used in the RELION particle subtraction routine to obtain 86,644 particles representing that region. The segmented map was filtered to 15 Å and used to generate a reference and a mask (fig. S18B, cerulean surface and grey mesh, respectively) for an overall 3D refinement and post-processing (fig. S18B, grey surfaces) using 86,644 subtracted particles. The densities corresponding to RLCs were insufficient to accommodate the RLC molecules (fig. S18C, inserts, light green ribbons), presumably due to RLC disorder, since the RLCs were unresolved in both the N and C lobes (fig. S18D, black arrows). Therefore, we used the overall 3D map, filtered to 15 Å, as a reference and a mask (fig. S18E, dark green surface and grey mesh, respectively) for the unsupervised sorting of the subtracted particles. The resultant class averages and corresponding frequencies for the three classes are shown in fig. S18F: class 1 (n=42,634) (fig. S19F, green surface), class 2 (n=17,133) (fig. S18F, chartreuse surface), and class 3 (n=26,877) (fig. S18F, teal surface). Classes 2 and 3 were discarded because they lacked a large portion of the RLC density. Class 1 was used for 3D refinement, using the 3D class-average low-pass filtered to 15 Å as the starting reference and mask (fig. S18G, green surface and grey mesh, respectively). The 3D reconstruction of Class1 RLCs showed that RLC densities were still incomplete (fig. S18H, inserts, light green ribbons) since the N and C lobes remained partially unresolved (fig. S18I, black arrows). The particles from class 1 (n=42,634) were used as the input for the second iteration of unsupervised classification. The class average was filtered to 15 Å and used as the reference and mask (fig. S18J, dark red surface and grey mesh, respectively). The resulting class averages and corresponding frequencies from the 3-class sorting are shown in fig. S18K: class 1 (n=10,625) (red surface), class 2 (n=29,660) (dark orange surface), and class 3 (n=2,349) (orange surface). Classes 1 and 3 were discarded due to partially missing RLC densities,

while class 2 was low-pass filtered to 15 Å and used as the starting reference and mask (fig. S18L, dark orange surface and grey mesh, respectively) for the 3D refinement. In the resultant map, the trailing RLC was semi-disordered (fig. S18M, inserts, light green ribbons), since its N and C lobes were not fully resolved (fig. S18N, black arrows), while the leading RLC was resolved. The particles from class 2 (n=29,660) were used as the input for the third iteration of unsupervised sorting. The class average was again filtered to 15 Å and used as the reference and mask (fig. S18O, navy blue surface and grey mesh, respectively). The resulting class averages from the 3-class sorting are shown in fig. S18P: class 1 (n=2,192) (blue surface), class 2 (n=1,265) (cyan surface), and class 3 (n=26,203) (light blue surface). The class 3 class-average was low-pass filtered to 12 Å and used as the starting reference and mask (fig. S18Q, light blue surface and grey mesh) for 3D refinement. The analysis of the RLC densities (fig. S18R, insert, light green ribbons) showed that the C-lobe of the trailing head was fully resolved, but the N-lobe was partially missing (fig. S18S, black arrow). The last unsupervised sorting was started from the low-pass-filtered class average as the reference and the mask (fig. S18T, deep pink surface and grey mesh, respectively). The class output maps from the final 3-class sorting are shown in fig. S18U: class 1 (n=8,153) (magenta surface), class 2 (n=175) (hot pink surface), and class 3 (n=17,875) (light pink surface). The 3D refinement was performed using the same approach as described previously, with the class-average filtered to 15 Å as the model and mask (fig. S18V, light pink surface and grey mesh). The final map showed the secondary structures of both trailing and leading RLCs (fig. S18W, inserts) and was used to generate an atomic model of the HMM that included the neck region (fig. S18X, green ribbons). Given the high heterogeneity of the RLCs and the limited initial class sizes for myosin bound to actin molecules 5–6, 2–3, and 1–2, we chose not to pursue further classification of the RLCs in these subsets.

Regulatory light chain (RLC) modeling: The RLC from the thick filament PDB 8G4L (*6*) was used as a template for the porcine cardiac ventricular amino acid sequence (UniProt: Q8MHY0) in SWISS MODEL (*87*), as they share 96.39% identity. To build the HMM model with RLCs, the final map from unsupervised sorting (fig. S18W) was used as input. The trailing and leading heads were independently segmented from the map filtered to 6 Å using the UCSF Chimera (*73*) segmenting tool (*82*), and auto-sharpened using PHENIX (*76*). The two models were rigid-body docked into the two sharpened maps and refined in Namdinator (*74*). The high-resolution model of myosin motor domains and ELCs from Class 6-7, which yielded a perfect fit to the 6 Å map, was annealed with the rod domain, and RLC coordinates obtained from the 6 Å map using the PHENIX (*76*) rebuild algorithm were used to reconnect residues 808 and 809. The parameters for the resultant model are shown in Table S2.

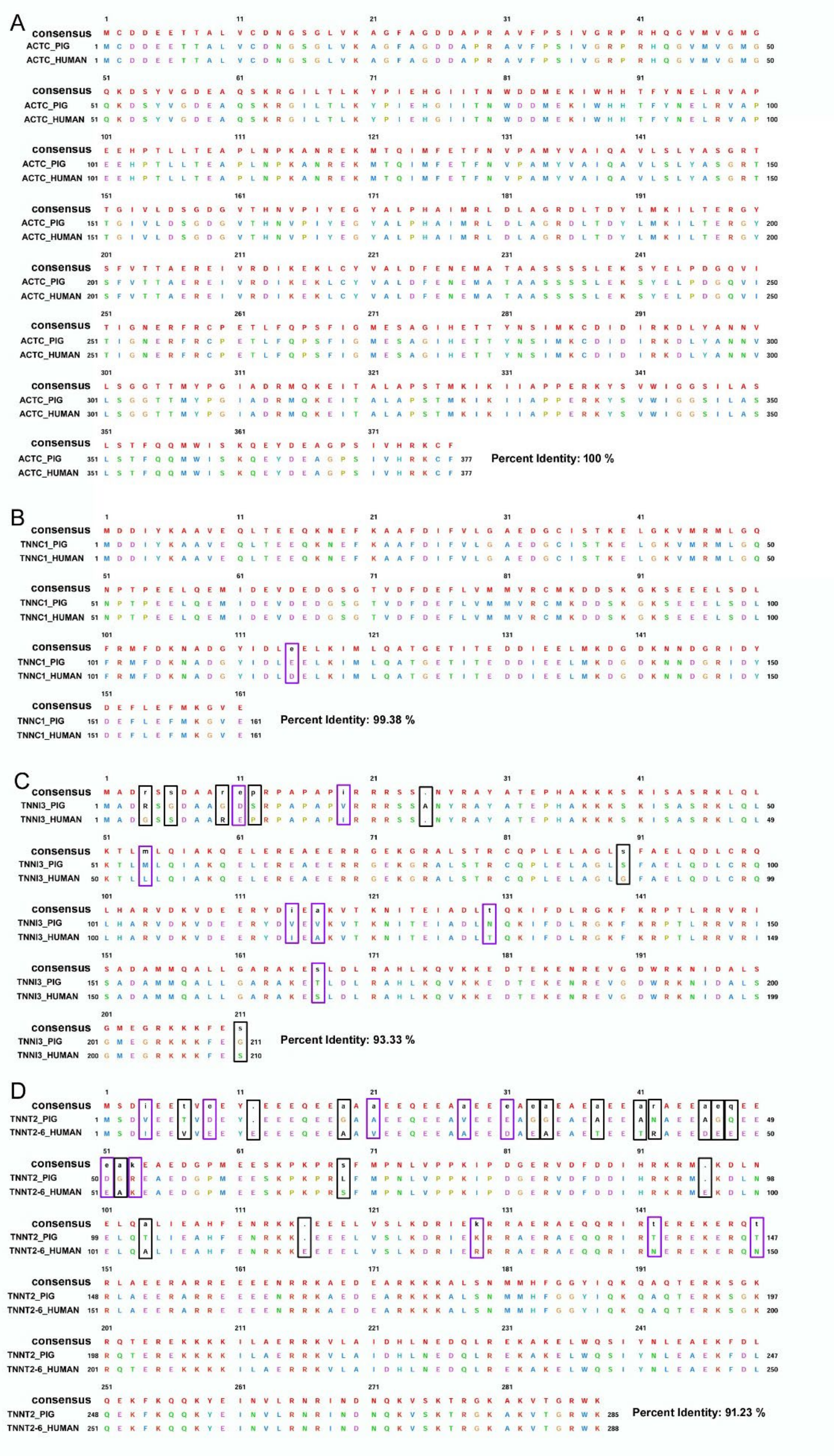

A
consensus
ACTC_PIG
ACTC_HUMAN
Percent Identity: 100 %
B
consensus
TNNC1_PIG
TNNC1_HUMAN
Percent Identity: 99.38 %
C
consensus
TNNI3_PIG
TNNI3_HUMAN
Percent Identity: 93.33 %
D
consensus
TNNT2_PIG
TNNT2-6_HUMAN
Percent Identity: 91.23 %

Figure S1. Amino acid alignment of porcine and human cardiac actin, TnC, TnI, and TnT. (**A**) Sequence alignment of porcine and human cardiac actin, which share 100% identity. (**B**) Sequence alignment of porcine and human cardiac TnC. There is one conserved substitution between the porcine and human isoforms, indicated with a purple rectangle. (**C**) Amino acid alignment of porcine and human cardiac TnI. Differences between the porcine and human sequences are marked with black and purple rectangles, respectively, for non-conserved and conserved substitutions. (**D**) Amino acid alignment of porcine and human cardiac TnT. Differences between the porcine and human sequences are marked the same as in (C). Each alignment shows a consensus sequence (top) calculated using UCSF Chimera (*73*).

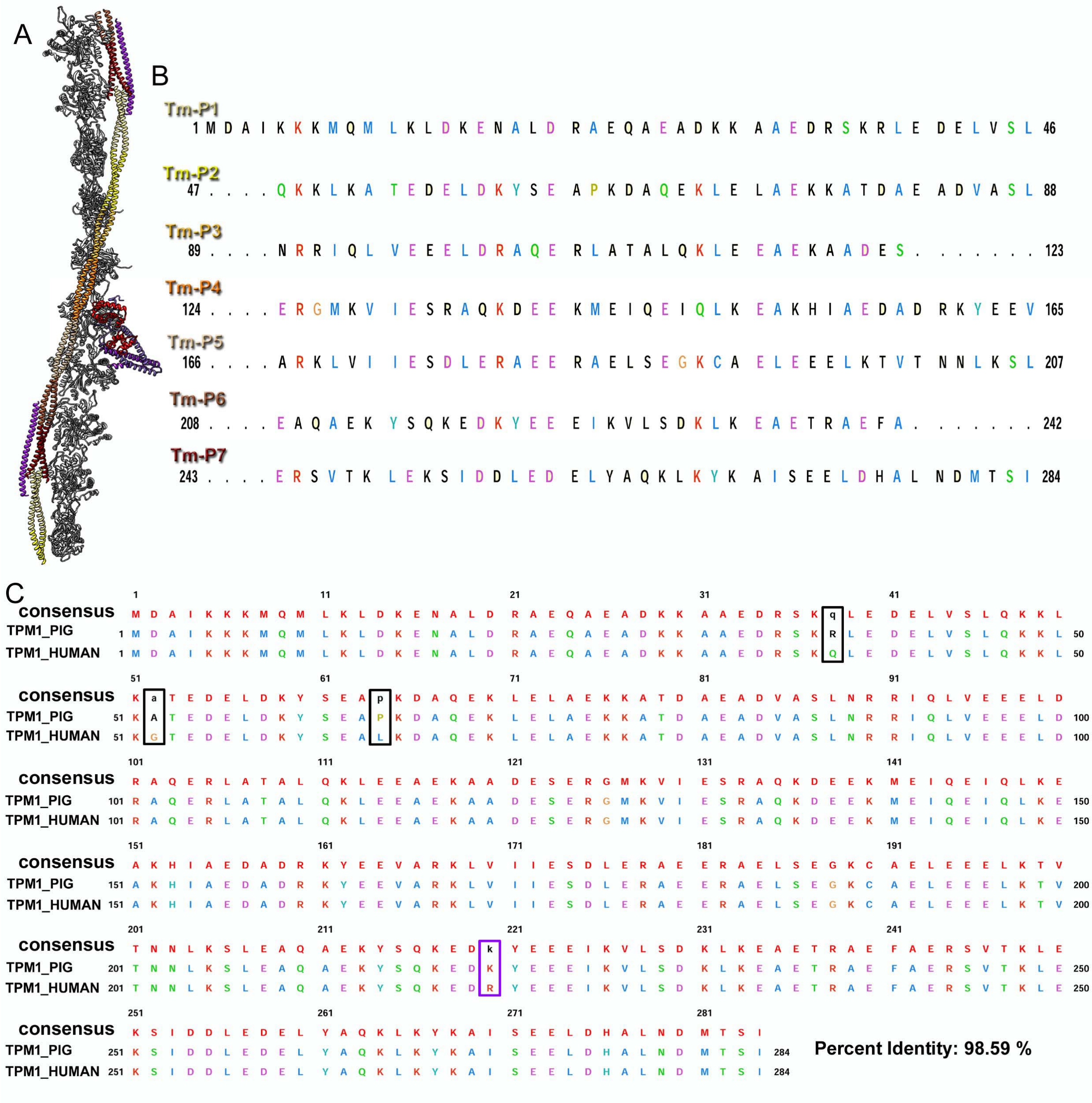

Figure S2. Amino acid alignment of porcine and human cardiac α-Tm. (**A**) Structure of the cTF RU, using the same color codes as main Fig. 1. The components of the RU are actin (gray ribbons), TnT (purple ribbons), TnI (medium purple ribbons), TnC (red ribbons), and Tm (multicolored ribbon). The seven periods of Tm, starting with period 1, are shown as ribbons in the following color order: khaki, yellow, gold, orange, tan, sienna, and dark red. (**B**) Canonical sequence alignment of the seven period repeats of porcine cardiac Tm. (**C**) Sequence alignment of

α-Tm from porcine and human species. Non-conserved differences are marked by a black rectangle, and conserved substitutions between porcine and human α-Tm are marked with a purple rectangle. Each alignment shows a consensus sequence (top) calculated using UCSF Chimera (*73*).

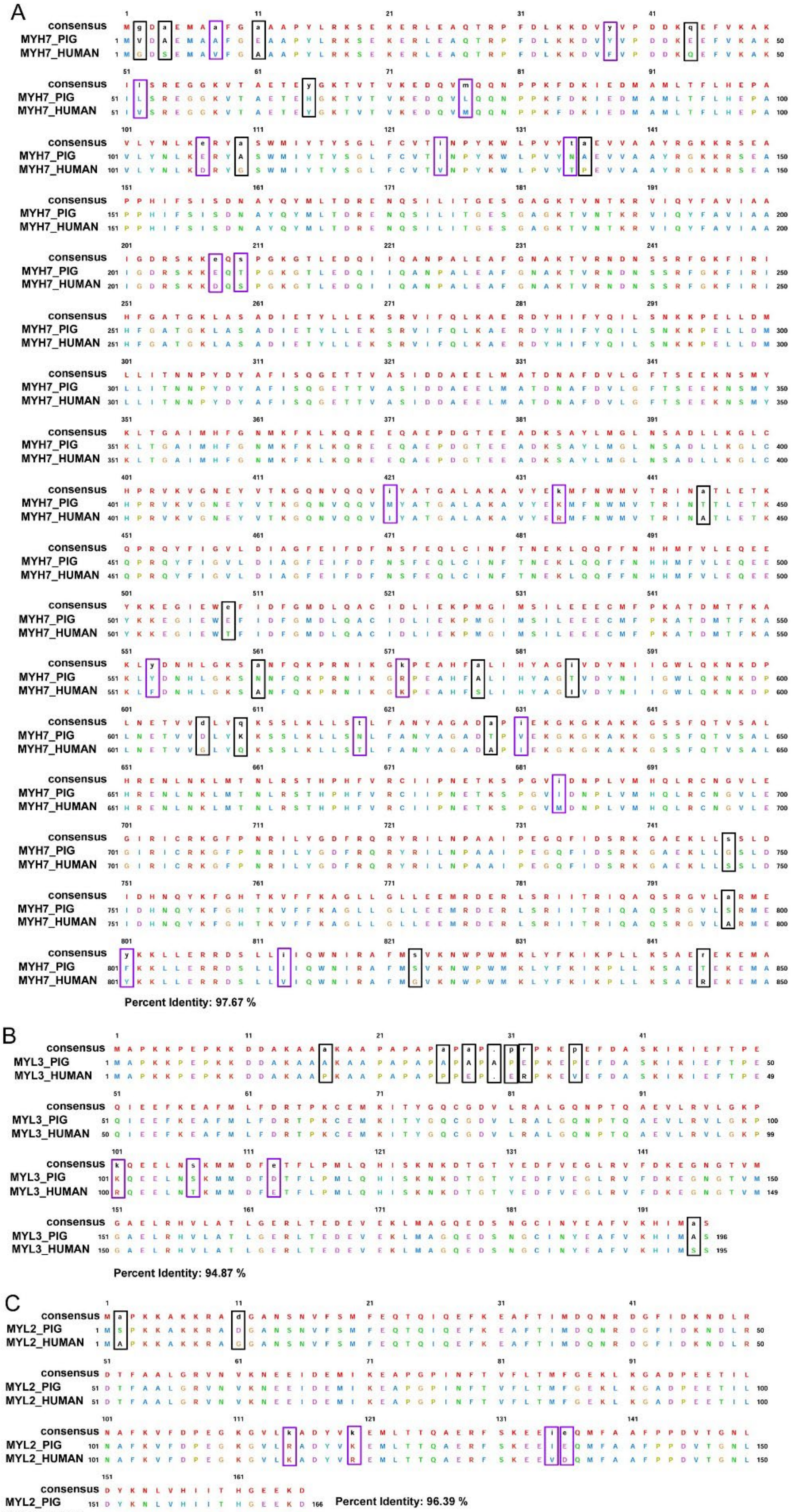

A
consensus
MYH7_PIG
MYH7_HUMAN
Percent Identity: 97.67 %
B
consensus
MYL3_PIG
MYL3_HUMAN
Percent Identity: 94.87 %
C
consensus
MYL2_PIG
MYL2_HUMAN
Percent Identity: 96.39 %

Figure S3. Amino acid alignment of porcine and human cardiac myosin and essential and regulatory light chains. (**A**) Sequence alignment of porcine and human cardiac myosin (residues 1-850). Differences between the porcine and human sequences are marked with black and purple rectangles, respectively, for non-conserved and conserved substitutions. (**B**) Sequence alignment of porcine and human cardiac ventricular essential light chain. Differences between the porcine and human sequences are marked the same as in (A). (**C**) Amino acid alignment of porcine and human cardiac ventricular regulatory light chains. Differences between the porcine and human sequences are marked the same as in (A) and (B). Each alignment shows a consensus sequence (top) calculated using UCSF Chimera (*73*).

cryo-EM

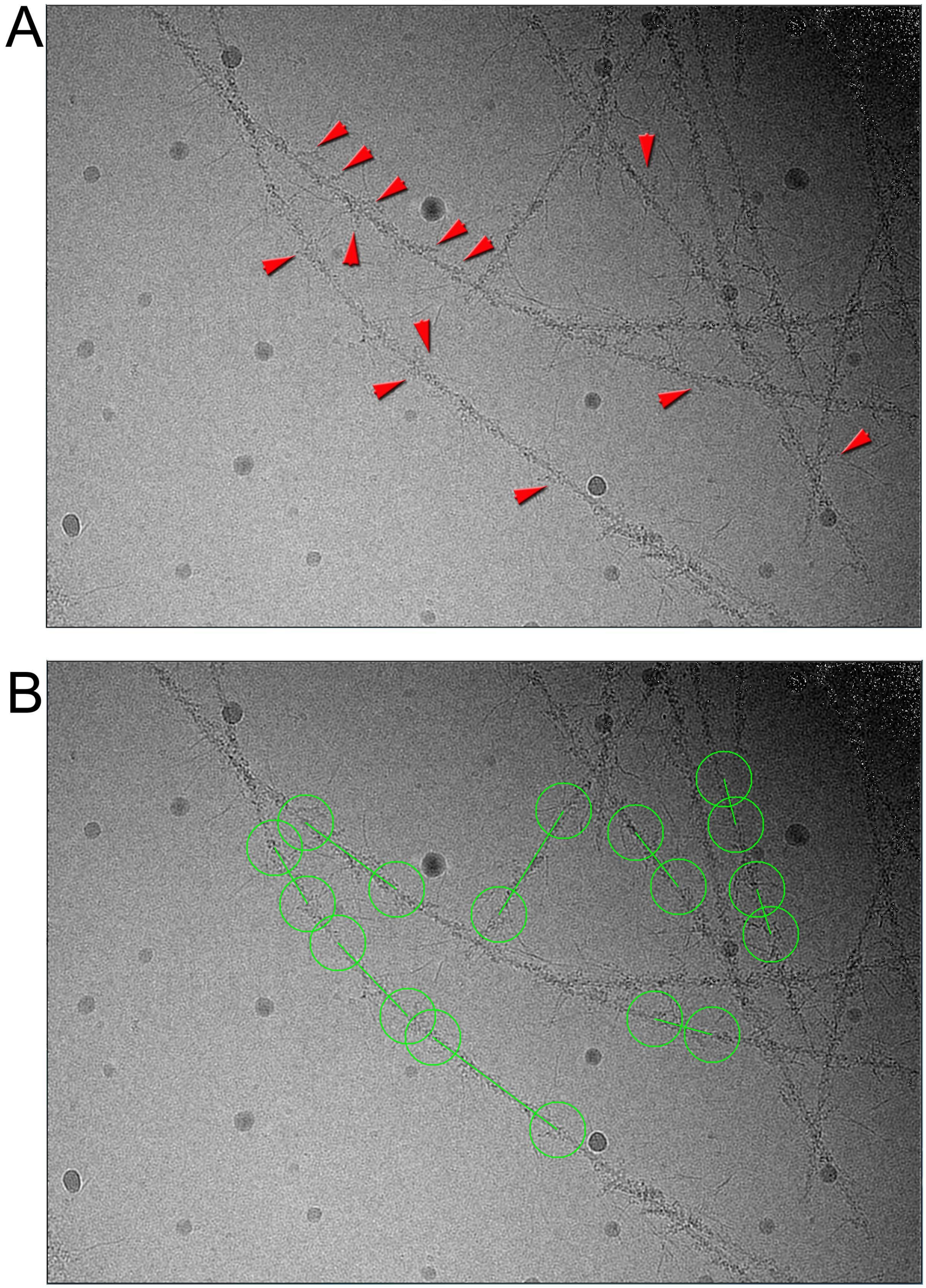

Figure S4. Cryo-EM of cardiac native TFs decorated with HMM at pCa=4. (**A**) Example cryo-EM micrograph of HMM-decorated cTFs. HMM molecules were clearly visible (red arrows), and areas of sparse decoration were used for manual selection. (**B**) Overlapping HMM-bound cTF segments were manually selected and extracted from the cryo-EM micrographs for 3D classification.

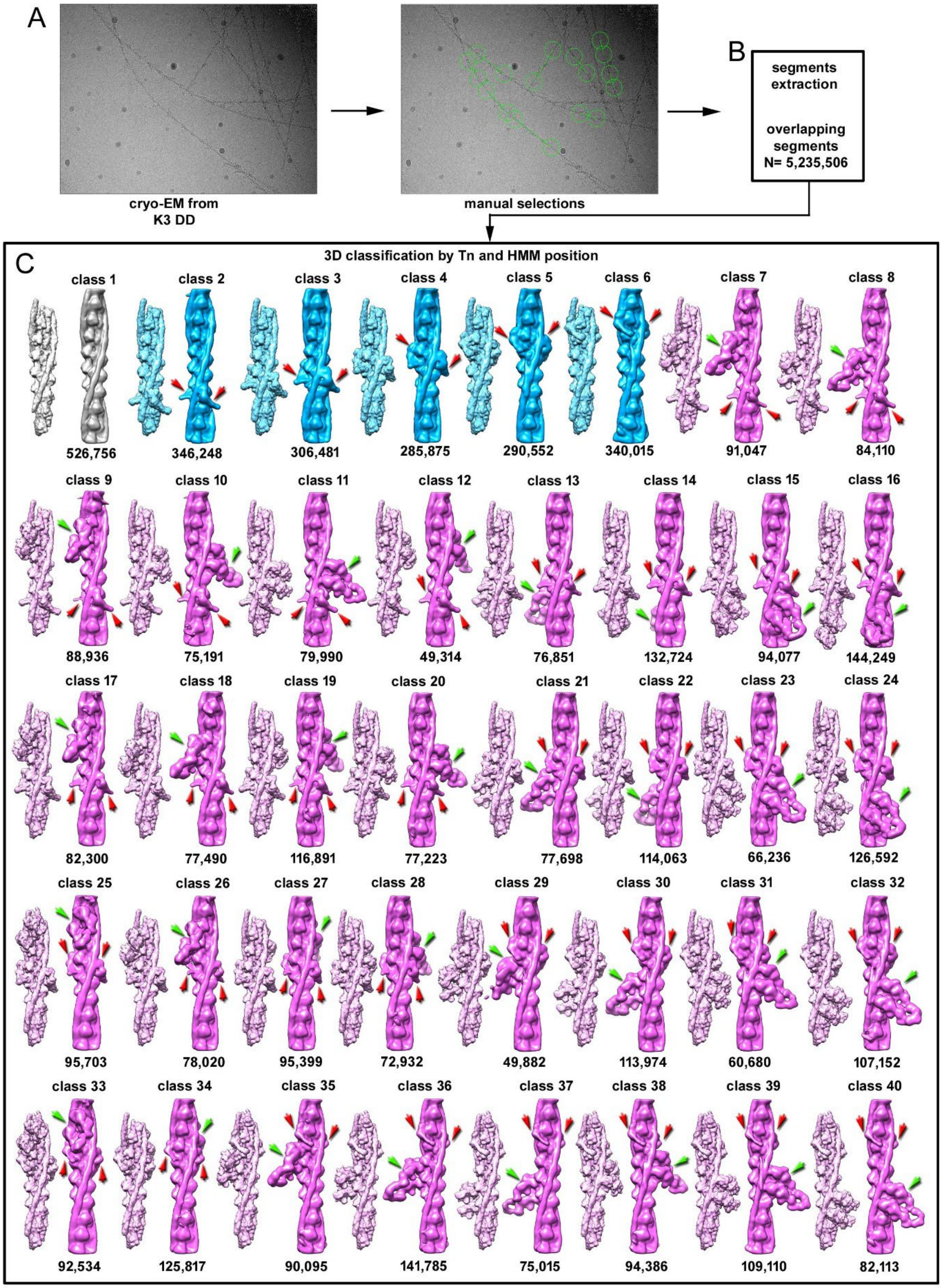

A
cryo-EM from
K3 DD
manual selections
B
segments
extraction
overlapping
segments
N= 5,235,506
C
3D classification by Tn and HMM position
class 1
526,756
class 2
346,248
class 3
306,481
class 4
285,875
class 5
290,552
class 6
340,015
class 7
91,047
class 8
84,110
class 9
88,936
class 10
75,191
class 11
79,990
class 12
49,314
class 13
76,851
class 14
132,724
class 15
94,077
class 16
144,249
class 17
82,300
class 18
77,490
class 19
116,891
class 20
77,223
class 21
77,698
class 22
114,063
class 23
66,236
class 24
126,592
class 25
95,703
class 26
78,020
class 27
95,399
class 28
72,932
class 29
49,882
class 30
113,974
class 31
60,680
class 32
107,152
class 33
92,534
class 34
125,817
class 35
90,095
class 36
141,785
class 37
75,015
class 38
94,386
class 39
109,110
class 40
82,113

Figure S5. Initial classification of HMM-decorated cTF. (**A** to **B**) Overlapping cTF segments were manually selected and extracted from cryo-EM micrographs for 3D classification (n = 5,235,506). (**C**) The extracted segments were sorted using a supervised method. Reference surfaces for sorting are shown as short maps in light gray (actin with Tm), light blue (TF without HMM), and light pink (TF with HMM is all possible positions). Of note, references lacked RLCs. The resulting class averages are shown next to the models as long surfaces using the following color codes: actin with Tm only (gray surface), TF without HMM bound (cerulean surfaces, Tn cores are marked with red arrows), TFs with HMM bound to different positions in regard to Tn core (pink surfaces; Tn cores indicated by red arrows, and HMM indicated by green arrows). The goal was to select segments containing the HMM molecule near the particle center (to avoid RLC clipping) that represent all available HMM positions on the RU. Particles from Class 10 (representing HMM bound to actins 1 and 2l Class 1-2), Class 11 (HMM bound to actins 2 and 3; Class 2-3), Class 31 (HMM bound to actins 5 and 6; Class 5-6), Class 39 (HMM bound to actins 6 and 7; Class 6-7), and Class 37 (HMM bound to actins 7 and 1; Class 7-1) were extracted for further processing depicted in figs. S7 to S15, and S18.

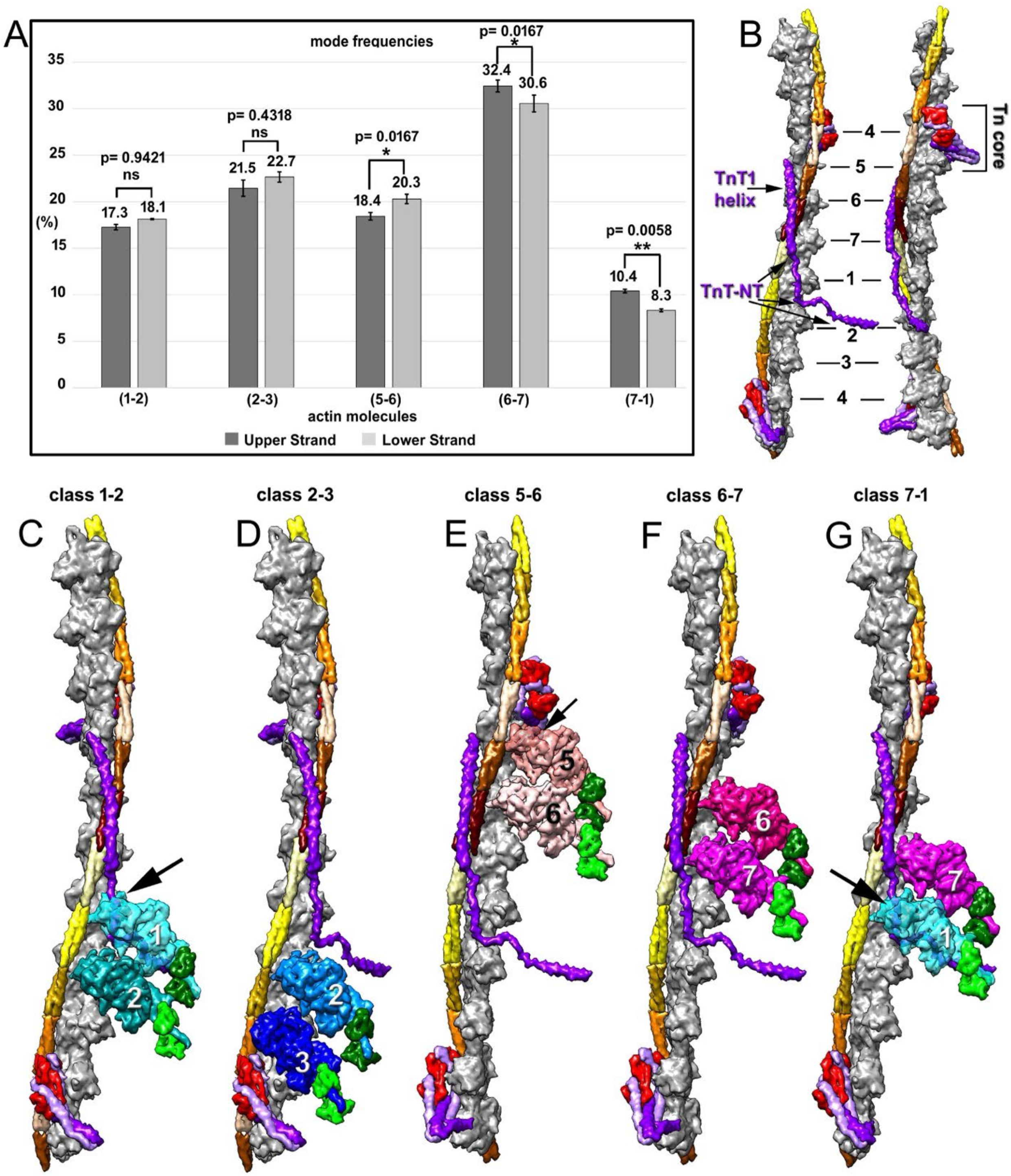

Figure S6. Hypothetical model of TnT restricting access of myosin heads to actins 1 and 5 of the TF RU. (**A**) Frequency distribution of HMM bound to actin molecules 1-2, 2-3, 5-6, 6-7, and 7-1 on the upper and lower strands. Statistical differences between structural classes were assessed

using two-way ANOVA and Holm-Šídák's test (data plotted from tables S5-S6). Significance is denoted by asterisks: n.s., not significant; *$P \leq 0.05$; **$P \leq 0.01$. (**B**) Model of the cardiac TF using the same color code as Fig. 1. Actin molecules are numbered along the RU (black numbers). The full-length TnT is represented as a purple surface; however, the exact location of the N' and C' termini is hypothetical. (**C** to **G**) The hypothetical interference of TnT with myosin heads bound to actins 1-2, 2-3, 5-6, 6-7, and 7-1 is denoted with black arrows. (C) Class 1-2 trailing myosin head (cyan surface) is likely to have a major clash with the N-terminus of TnT (purple surface) at actin 1 (large black arrow). (E) Class 5-6 trailing myosin head (salmon surface) may have a minor clash with the Tn-core (small black arrow) at actin 5. (G) Class 7-1 myosin leading head (cyan surface) should have a clash with TnT at actin 1 (large black arrow), consistent with the low frequency of class 7-1.

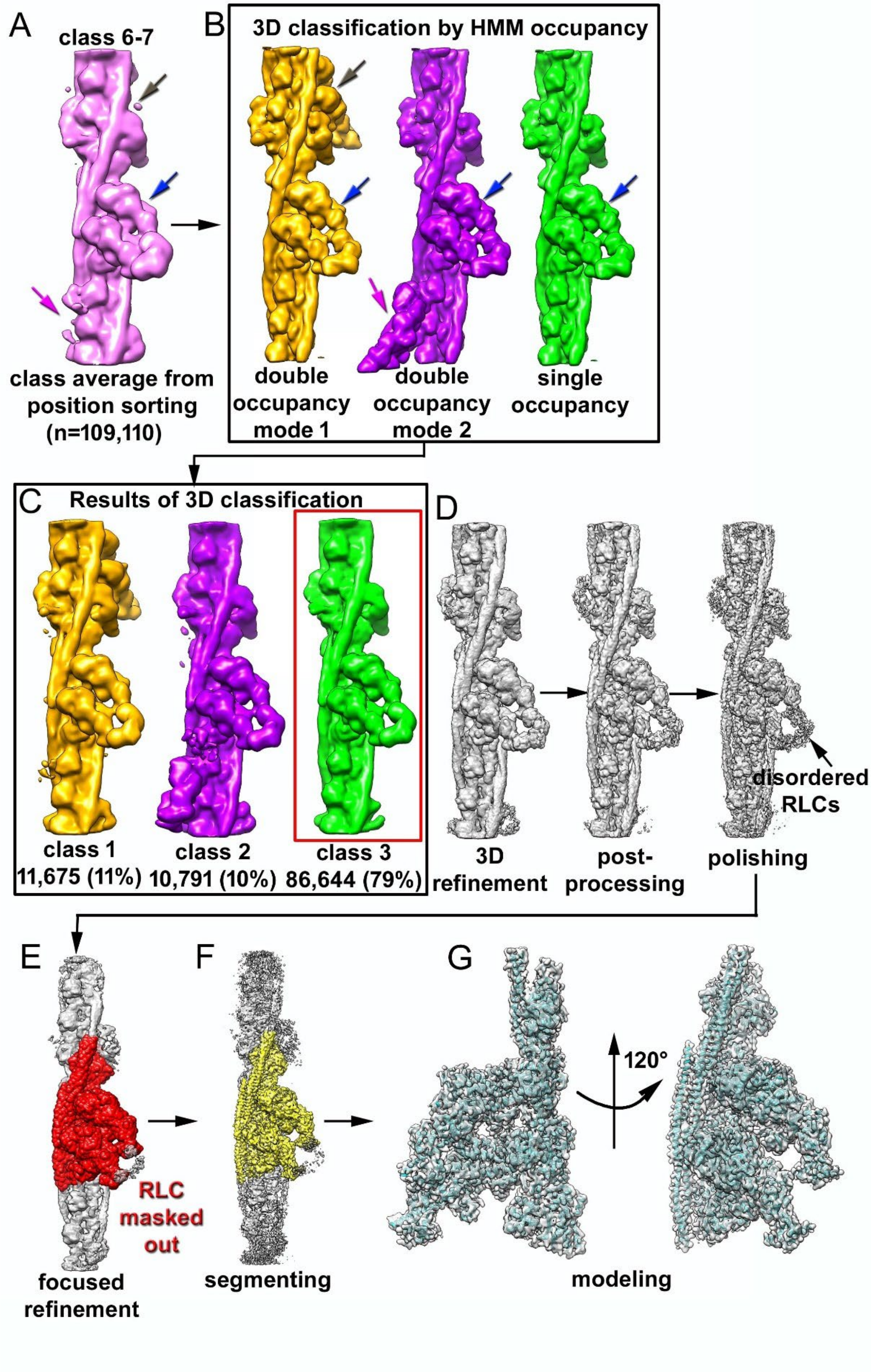

A
class 6-7
class average from position sorting (n=109,110)
B
3D classification by HMM occupancy
double occupancy mode 1
double occupancy mode 2
single occupancy
C
Results of 3D classification
class 1 11,675 (11%)
class 2 10,791 (10%)
class 3 86,644 (79%)
D
3D refinement
post-processing
polishing
disordered RLCs
E
RLC masked out
focused refinement
F
segmenting
G
120°
modeling

Figure S7. 3D sorting and refinement of Class 6-7. (**A**) The class 39 average (fig. S5C), shown as a hot pink surface, guided the construction of two templates that incorporated a second bound HMM molecule, based on HMM density traces in the class 39 average map (blue, magenta, and brown arrows). (**B**) Three templates for supervised sorting: mode 1 (orange surface, brown and blue arrows), mode 2 (purple surface, magenta and blue arrows), and single-occupancy HMM (green surface, blue arrow). (**C**) Resultant class averages and corresponding frequencies. Segments containing a single HMM molecule near the filament's center (red box) were used for 3D refinement and Bayesian polishing (**D**). (**E**) A focused refinement with a 10 Å mask (red surface) improved resolution in the region where the HMM binds to actin subunits 6 and 7 of TF RU. The HMM region (**F**) was segmented from the final map (**G**) for modeling.

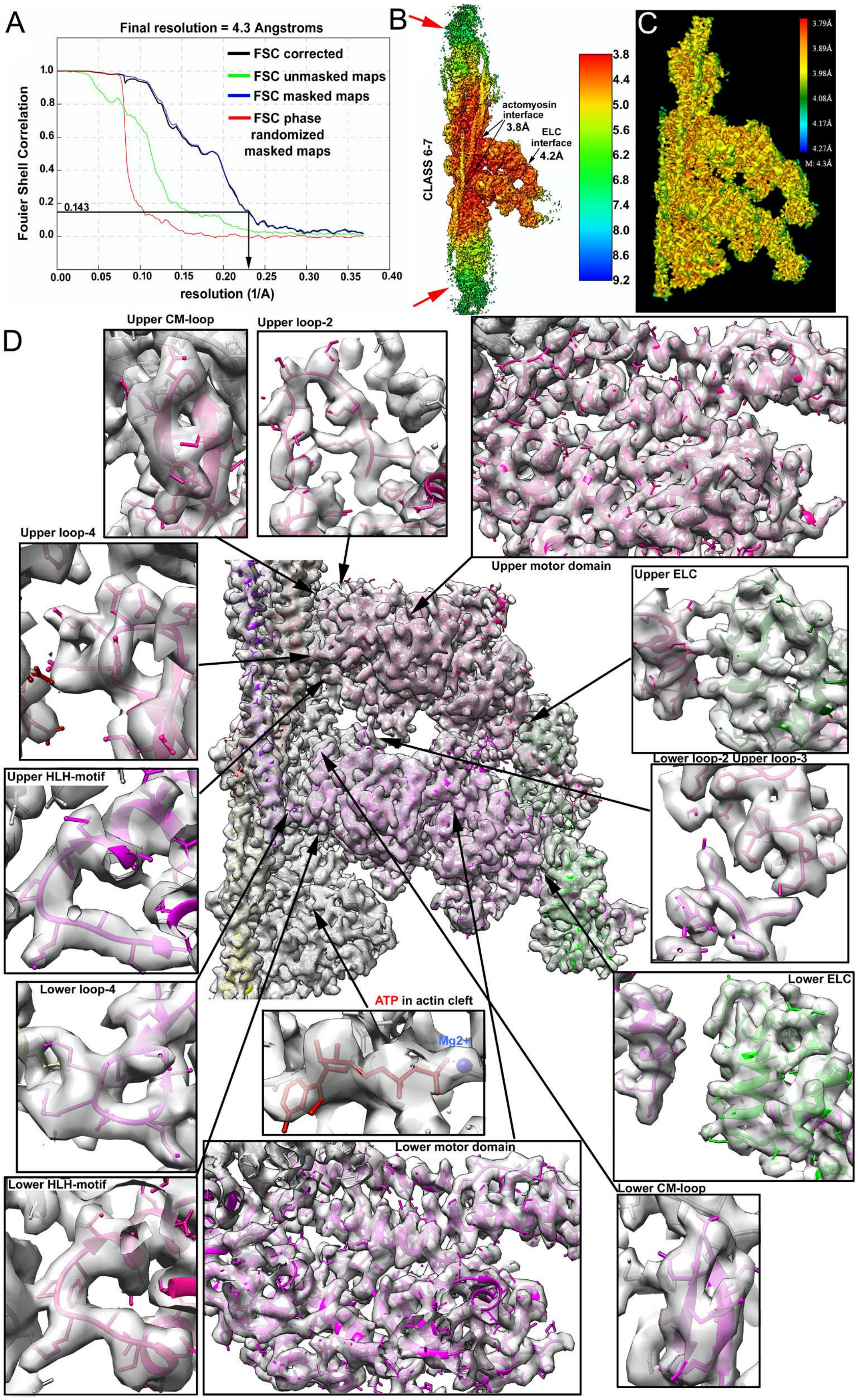

A
Final resolution = 4.3 Angstroms
FSC corrected
FSC unmasked maps
FSC masked maps
FSC phase randomized masked maps
Fouier Shell Correlation
resolution (1/A)
0.143
B
CLASS 6-7
actomyosin interface 3.8Å
ELC interface 4.2Å
C
M: 4.3Å
D
Upper CM-loop
Upper loop-2
Upper motor domain
Upper loop-4
Upper ELC
Upper HLH-motif
Lower loop-2 Upper loop-3
Lower loop-4
ATP in actin cleft
Mg2+
Lower ELC
Lower HLH-motif
Lower motor domain
Lower CM-loop

Figure S8. Resolution estimation for Class 6-7. (**A**) Fourier shell correlation (FSC) plot. The overall map resolution was calculated using the FSC criterion of 0.143. (**B**) Local resolution map determined by RELION implementation. The resolution is ~3.8 Å at the actomyosin interface and 4.2 Å at the ELC interface, while it drops to ~9.2 Å in the distal regions of the map (red arrows). (**C**) Local resolution estimation based on the final map using CryoRes. (**D**) 3D reconstruction of the HMM bound to actin molecules 6 and 7 is shown (grey surface) along with its corresponding model (colored atoms). Actin is shown in gray; period 1 of (Tm) is shown in khaki; period 2 is shown in yellow; period 6 is shown in sienna; and period 7 is shown in dark red. The TnT1 helix is shown in purple. The trailing myosin is deep pink, the trailing ELC is dark green, while the leading head is magenta, and the leading ELC is green. The high-resolution cryo-EM map reveals atomic details of the structural elements of the cardiac HMM involved in interactions with actin, Tm, ELC, and between trailing and leading myosin heads. Detailed views of the various components discussed in the paper are shown alongside the atomic model to illustrate the quality of the data.

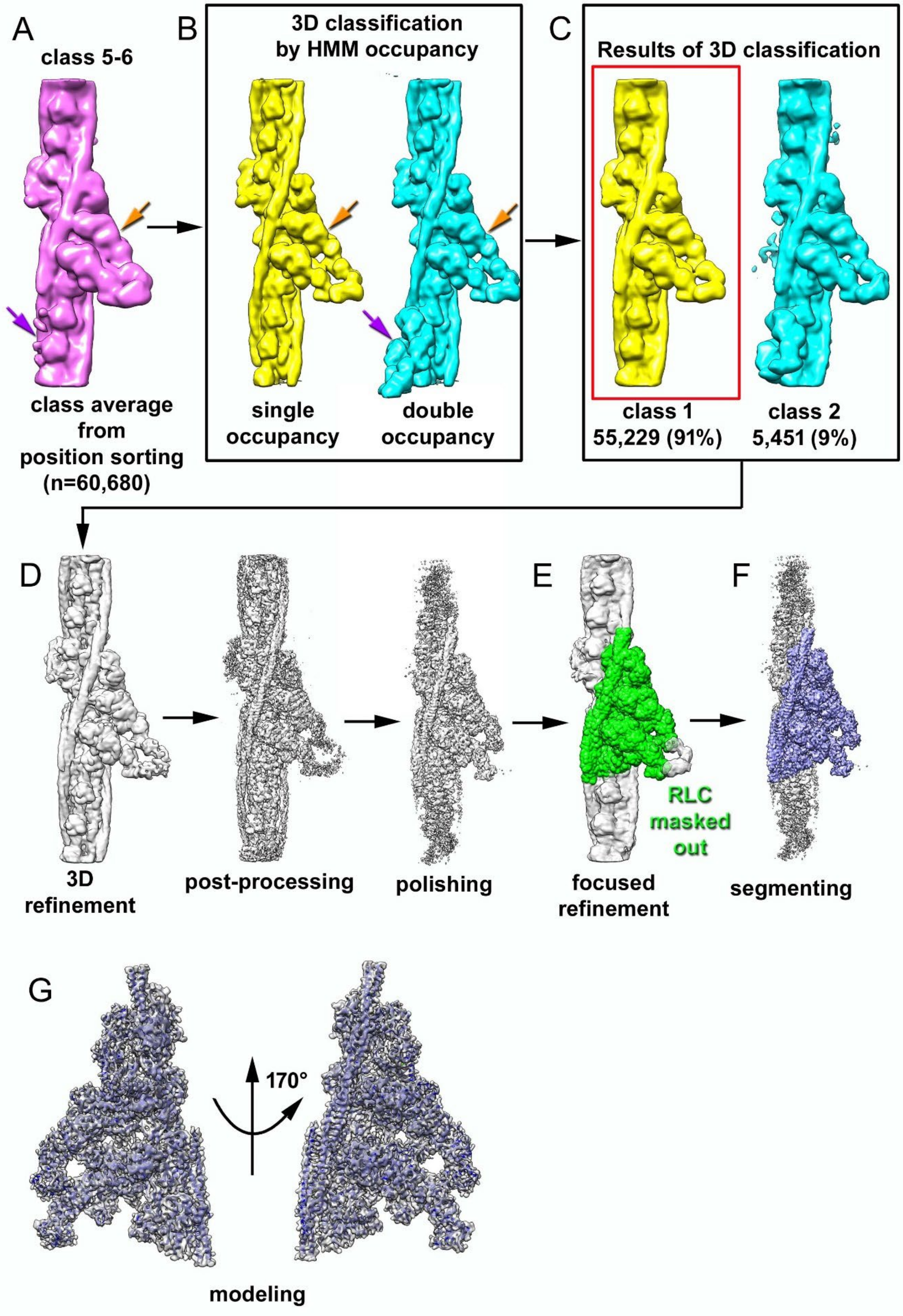
A
class 5-6
class average
from
position sorting
(n=60,680)
B
3D classification
by HMM occupancy
single
occupancy
double
occupancy
C
Results of 3D classification
class 1
55,229 (91%)
class 2
5,451 (9%)
D
3D
refinement
post-processing
polishing
E
focused
refinement
RLC
masked
out
F
segmenting
G
170°
modeling

Figure S9. 3D sorting and refinement of Class 5-6. (**A** and **B**) The class 31 average (fig. S5C), shown as a hot pink surface, guided the construction of two templates that incorporated either one or two bound HMM molecules, based on HMM density traces in the class 31 average map (purple and orange arrows) that are shown in (**B**) (yellow and cyan surfaces). (**C**) Resultant class averages and corresponding frequencies. Segments that contained one HMM molecule near the center of the filament (yellow surface, red box) were used for 3D refinement and Bayesian polishing (**D**). (**E**) A focused refinement with a 10 Å mask (green surface) was applied to improve resolution in the region where the HMM was bound to actin molecules 5 and 6. The HMM region was segmented (lavender surface) (**F**) from the final map (blue ribbons) (**G**) for modeling.

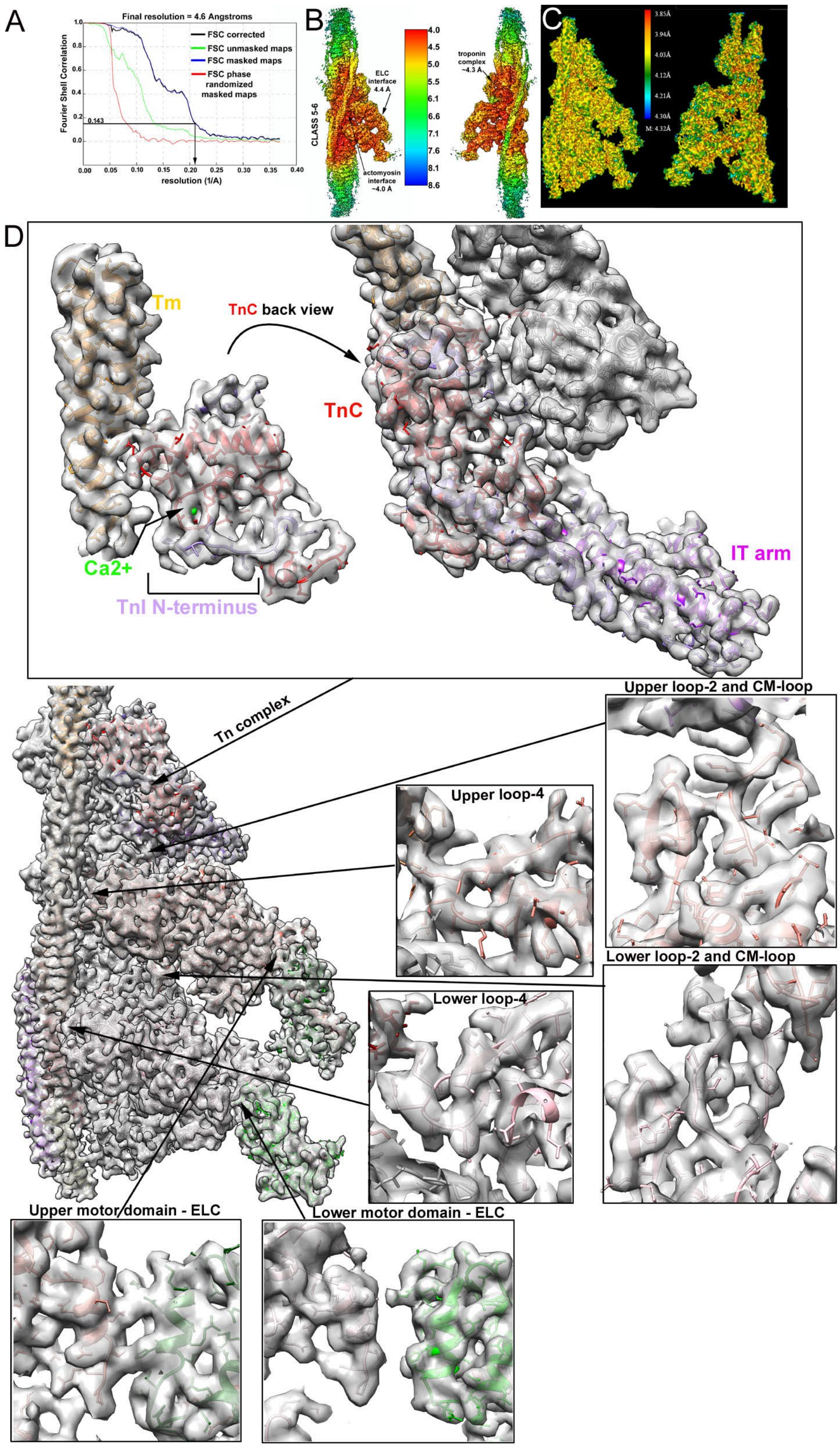

A
Final resolution = 4.6 Angstroms
Fourier Shell Correlation
resolution (1/A)
FSC corrected
FSC unmasked maps
FSC masked maps
FSC phase randomized masked maps
0.143
B
CLASS 5-6
ELC interface 4.4 Å
actomyosin interface ~4.0 Å
troponin complex ~4.3 Å
C
D
Tm
TnC back view
TnC
Ca2+
TnI N-terminus
IT arm
Tn complex
Upper loop-2 and CM-loop
Upper loop-4
Lower loop-2 and CM-loop
Lower loop-4
Upper motor domain - ELC
Lower motor domain - ELC

Figure S10. Resolution estimation for Class 5-6. (**A**) Fourier shell correlation (FSC) plot. The overall resolution of the map was calculated using the FSC criterion of 0.143. (**B**) Local resolution map determined by the RELION implementation. The resolution is ~4.0 Å at the actomyosin interface, 4.4 Å at the ELC interface, and ~4.3 Å at the Tn core. (**C**) Local resolution estimated from the final map using CryoRes. (**D**) 3D reconstruction of the HMM bound to actin molecules 5 and 6 is shown (gray surface) along with its corresponding model (colored atoms). The color code is the same as Fig. 1. The high-resolution cryo-EM map reveals atomic details of the structural elements of the cTF (i.e., Tn core, actin, and Tm) and of the cardiac HMM involved in interactions with actin, the Tn core, Tm, ELC, and between trailing and leading myosin heads. Detailed snapshots of the various components discussed in the paper are shown along with the atomic model.

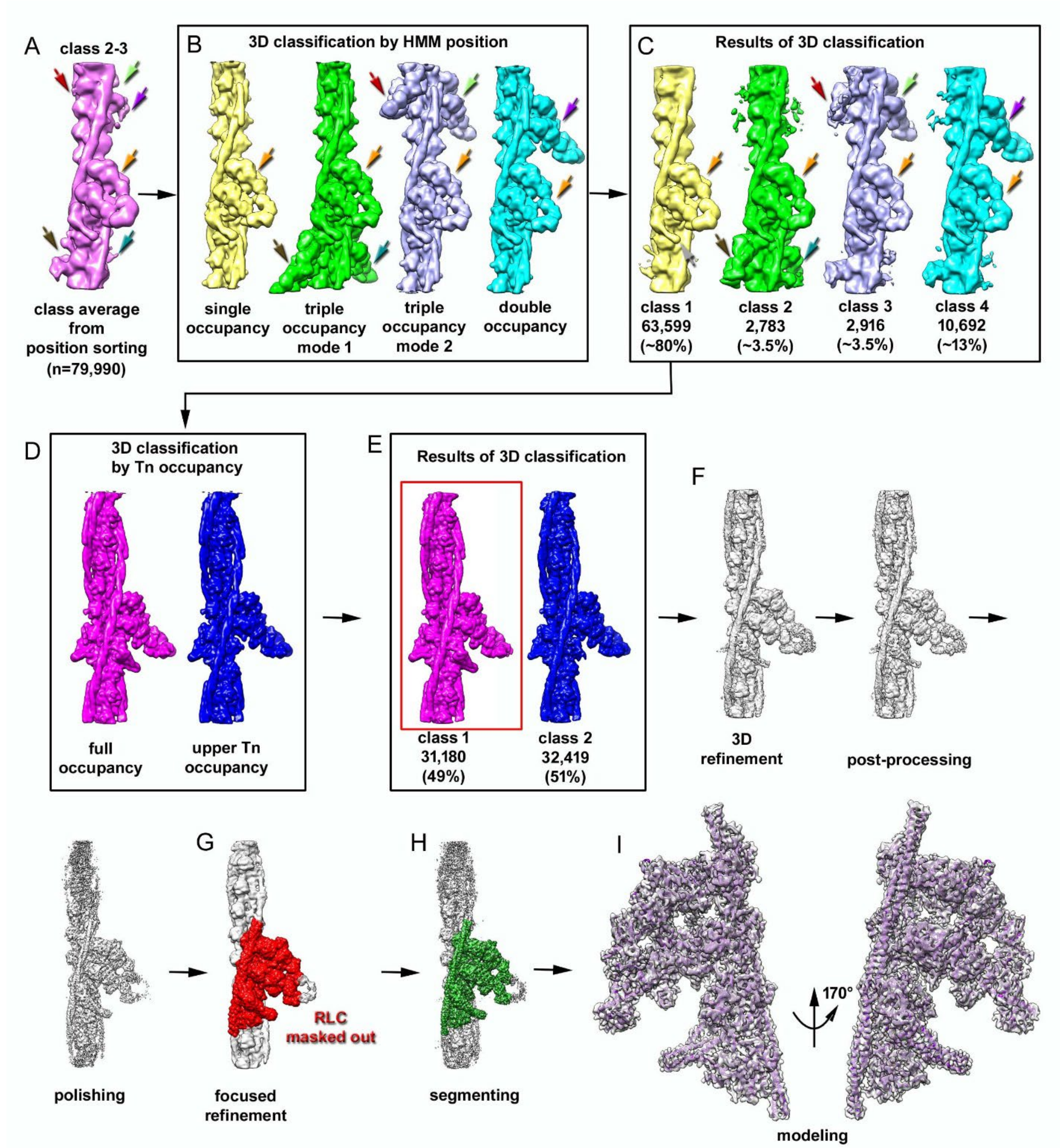

Figure S11. 3D sorting and refinement of Class 2-3. (**A**) The class 11 (fig. S5C) average from the initial position classification (depicted as a hot pink surface) was used as the guide to build templates for supervised sorting based on traces of additional HMM molecules bound (dark red, chartreuse, purple, orange, brown, and teal arrows). (**B**) The resultant four templates have one

(yellow surface), two (cyan surface), or three (green and lavender surfaces) HMM molecules bound. (**C**) Resultant class averages and corresponding frequencies. The segments that contained one HMM molecule near the center of the filament (yellow surface) had insufficient density for the Tn core (grey arrow) and were sorted based on the integrity of the Tn tandem (**D**) – one template had both Tn cores (magenta), whereas the other had one Tn complex missing (blue). (**E**) Resultant class averages where the intact Tn tandem (magenta surface, red box) was re-extracted at a scale of 1.356 Apx and used for 3D refinement and Bayesian polishing (**F**). (**G**) A focused refinement with a 10 Å mask (red surface) was used to improve the resolution in the region where HMM was bound to actin molecules 2 and 3. The HMM region (dark green surface) (**H**) was segmented from the final map for modeling (purple ribbons) (**I**).

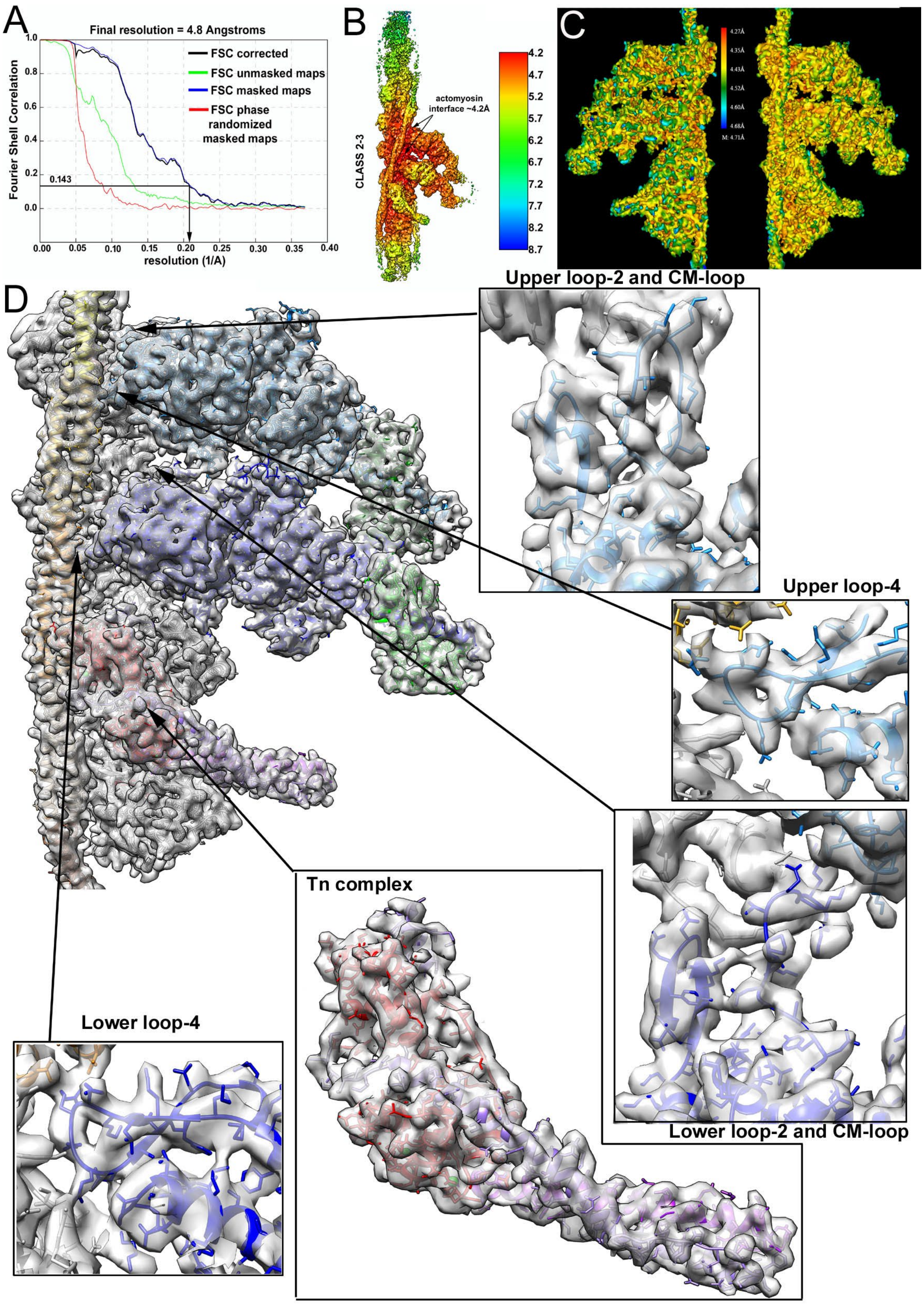

A
Final resolution = 4.8 Angstroms
FSC corrected
FSC unmasked maps
FSC masked maps
FSC phase randomized masked maps
Fourier Shell Correlation
0.143
resolution (1/A)
1.0
0.8
0.6
0.4
0.2
0.0
0.00
0.05
0.10
0.15
0.20
0.25
0.30
0.35
0.40
B
CLASS 2-3
actomyosin interface ~4.2Å
4.2
4.7
5.2
5.7
6.2
6.7
7.2
7.7
8.2
8.7
C
D
Upper loop-2 and CM-loop
Upper loop-4
Lower loop-2 and CM-loop
Tn complex
Lower loop-4

Figure S12. Resolution estimation for Class 2-3. (**A**) Fourier shell correlation (FSC) plot. The overall resolution of the map was calculated using the gold-standard FSC criterion of 0.143. (**B**) Local resolution map determined by RELION implementation. The resolution is ~4.2 Å at the actomyosin interface. (**C**) Local resolution calculated from the final map using CryoRes. (**D**) 3D reconstruction of the HMM bound to actin molecules 2 and 3 is shown (grey surface) along with its corresponding model (colored atoms). The color code is the same as Fig. 1. The cryo-EM map shows the atomic details of the structural elements of the cTF and cardiac HMM involved in interactions with actin, the Tn core, Tm, and ELC, as well as between trailing and leading myosin heads. Detailed views of the components discussed in the paper are shown alongside the atomic model.

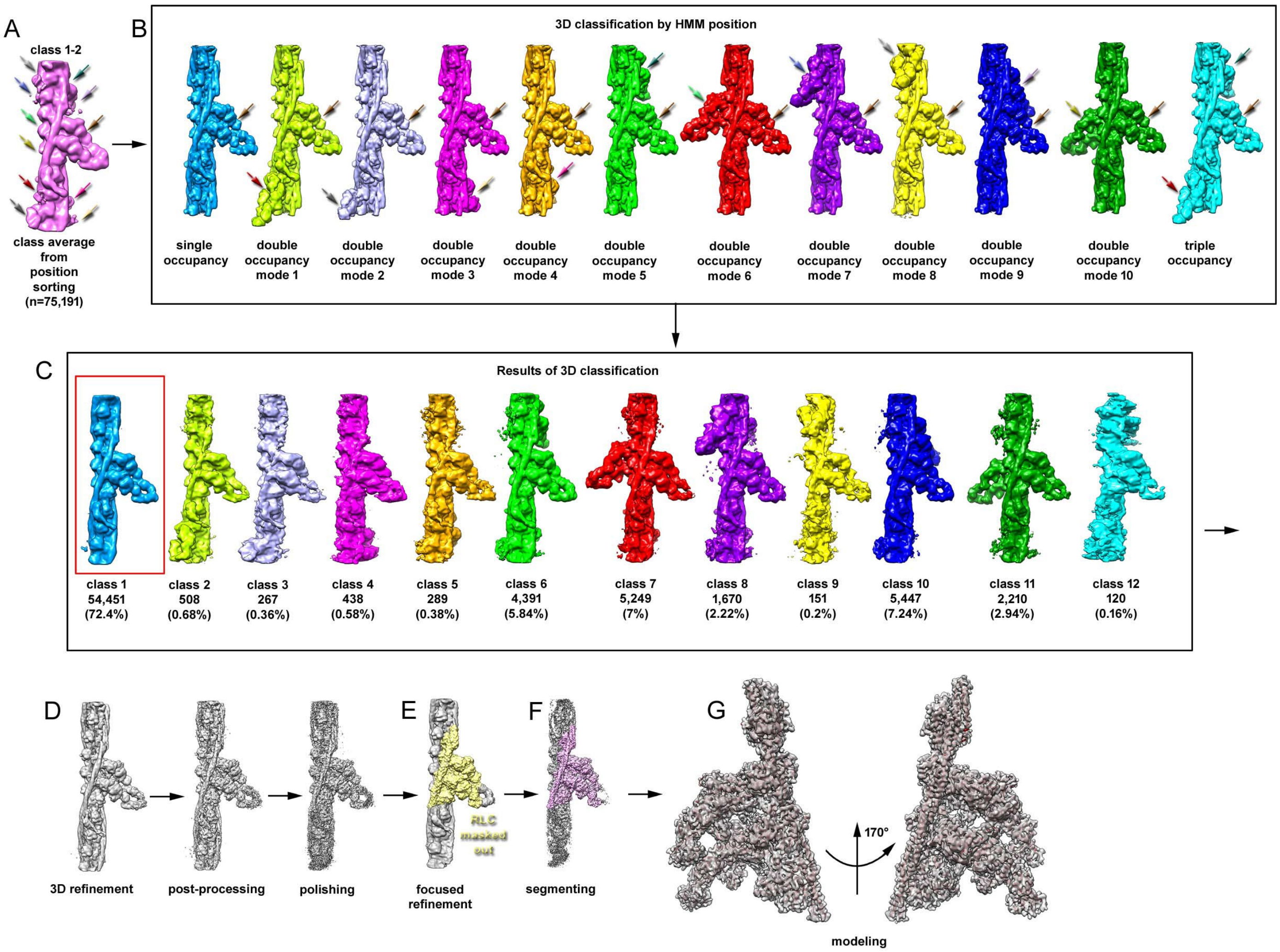

Figure S13. 3D sorting and refinement of Class 1-2. (**A**) The class 10 output map from the initial position classification (fig. S5C), shown as a hot pink surface, served as a guide for building templates with HMM bound to multiple positions, based on HMM traces revealed in the class 10 map (light gray, cornflower, light green, gold, dark red, dark gray, teal, light purple, sienna, deep pink, and khaki arrows). (**B**) Twelve templates were generated - one single-occupancy HMM (cerulean surface), ten double-occupancy modes (chartreuse, lavender, magenta, orange, green, red, purple, yellow, blue, and dark green surfaces), and one triple-occupancy HMM mode (cyan surface). (**C**) Resultant class averages and associated frequencies. Segments with one HMM molecule near the center of the filament (cerulean surface, red box) were re-extracted and used for

3D refinement and Bayesian polishing (**D**). (**E**) A focused refinement with the 10 Å mask (light yellow surface) was used to improve resolution in the region where HMM was bound to actin molecules 1 and 2. The HMM region was segmented (light pink surface) (**F**) from the final map for modeling (dark brown ribbons) (**G**).

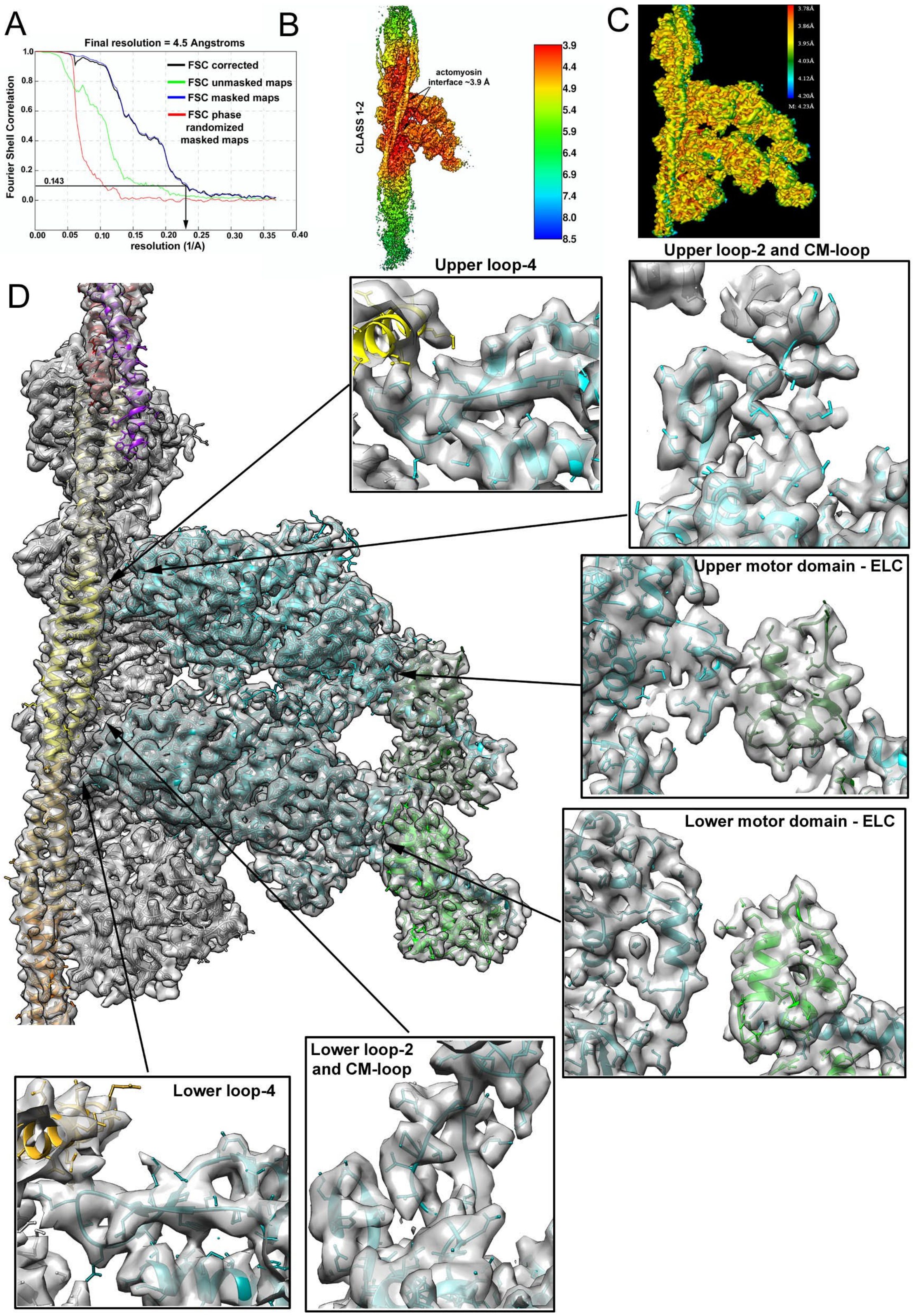

A
Final resolution = 4.5 Angstroms
FSC corrected
FSC unmasked maps
FSC masked maps
FSC phase randomized masked maps
Fourier Shell Correlation
0.143
resolution (1/A)
B
CLASS 1-2
actomyosin interface ~3.9 Å
C
D
Upper loop-4
Upper loop-2 and CM-loop
Upper motor domain - ELC
Lower motor domain - ELC
Lower loop-2 and CM-loop
Lower loop-4

Figure S14. Resolution estimation for class 1-2. (**A**) Fourier shell correlation (FSC) plot. The global resolution for the map was calculated using an FSC criterion of 0.143. (**B**) Local resolution map determined by RELION. The resolution is ~3.9 Å at the actomyosin interface. (**C**) Local resolution estimated from the final map using CryoRes. (**D**) 3D reconstruction of the HMM bound to actin molecules 1 and 2 is shown (gray surface) along with its corresponding model (colored atoms). The color code is the same as in Fig. 1. The high-resolution cryo-EM map reveals atomic details of the structural elements of the cardiac HMM that interact with cTF and ELC, as well as interactions between the trailing and leading myosin heads. Detailed views of the regions discussed in the paper are shown, along with the corresponding atomic model, to demonstrate the quality of the data.

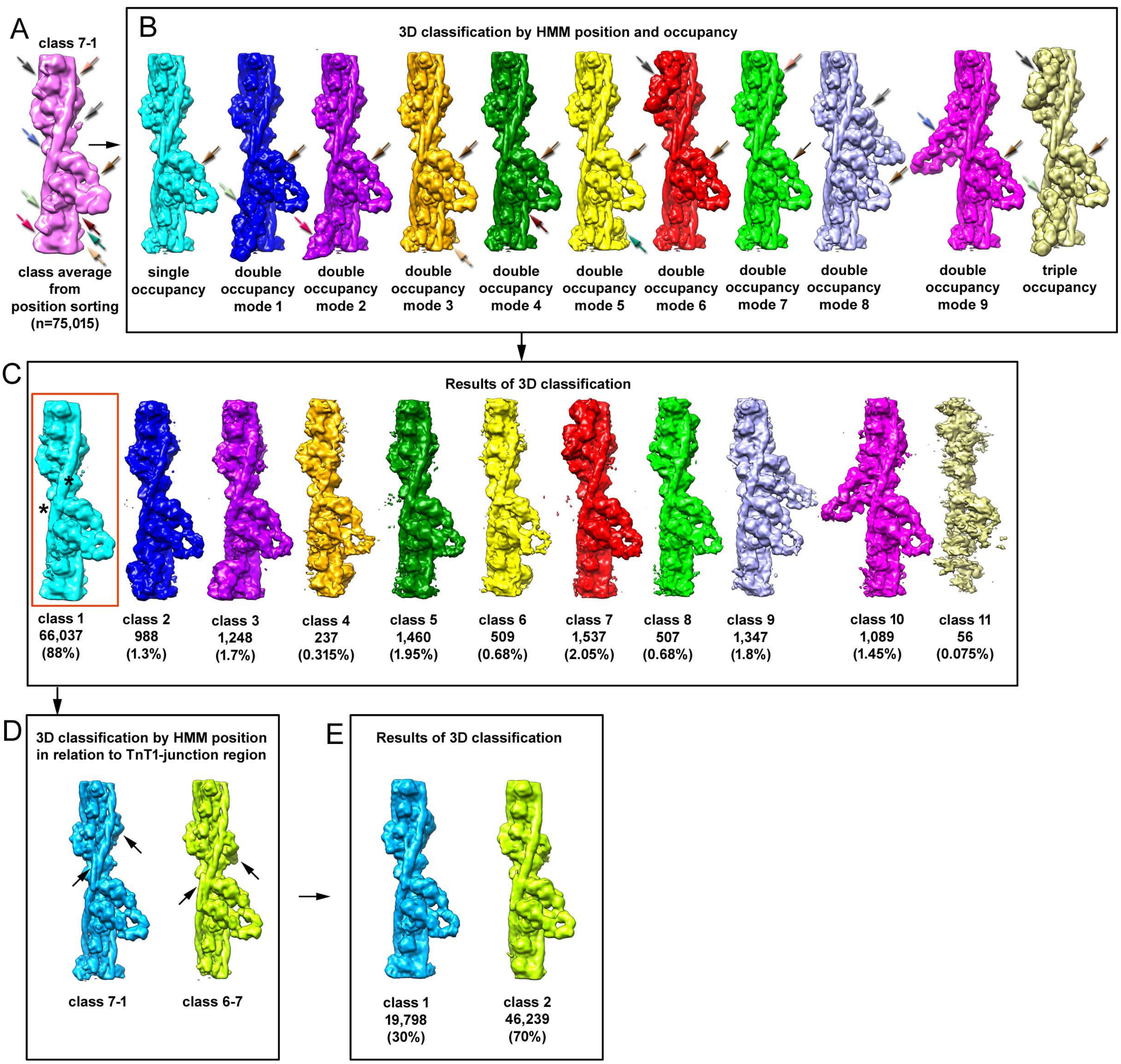

Figure S15. 3D sorting and refinement of class 7-1. (**A**) The class 37 output map from the initial position classification (fig. S5C), shown as a hot pink surface, was used as a guide to build templates with multiple HMM molecules bound, based on traces of additional HMM molecules (dark grey, cornflower, light green, deep pink, peach, light grey, sienna, dark brown, teal, and

khaki arrows) revealed in the class 37 class average. (**B**) The eleven templates used for supervised sorting included one single-occupancy HMM (cyan surface), nine double-occupancy modes (blue, purple, orange, dark green, yellow, red, green, lavender, and magenta surfaces), and one triple-occupancy mode (gold surface). (**C**) Resultant class averages and corresponding frequencies. Segments with one HMM molecule near the center of the filament (cyan surface, red box) were used for further classification based on the positioning of Tm junctions (black asterisks). Two junction regions indicated a mixture of HMM-bound modes. (**D**) Supervised sorting based on the position of HMM in relation to the Tm junction region, with HMM bound to actin molecules 7-1 (cerulean surface and black arrow) or 6-7 (chartreuse surface and black arrow). (**E**) Resultant class averages and corresponding frequencies. Class 1, representing segments with HMM at the position of actin molecule 7 from one RU and actin molecule 1 on the adjacent RU, accounts for only 30% of segments.

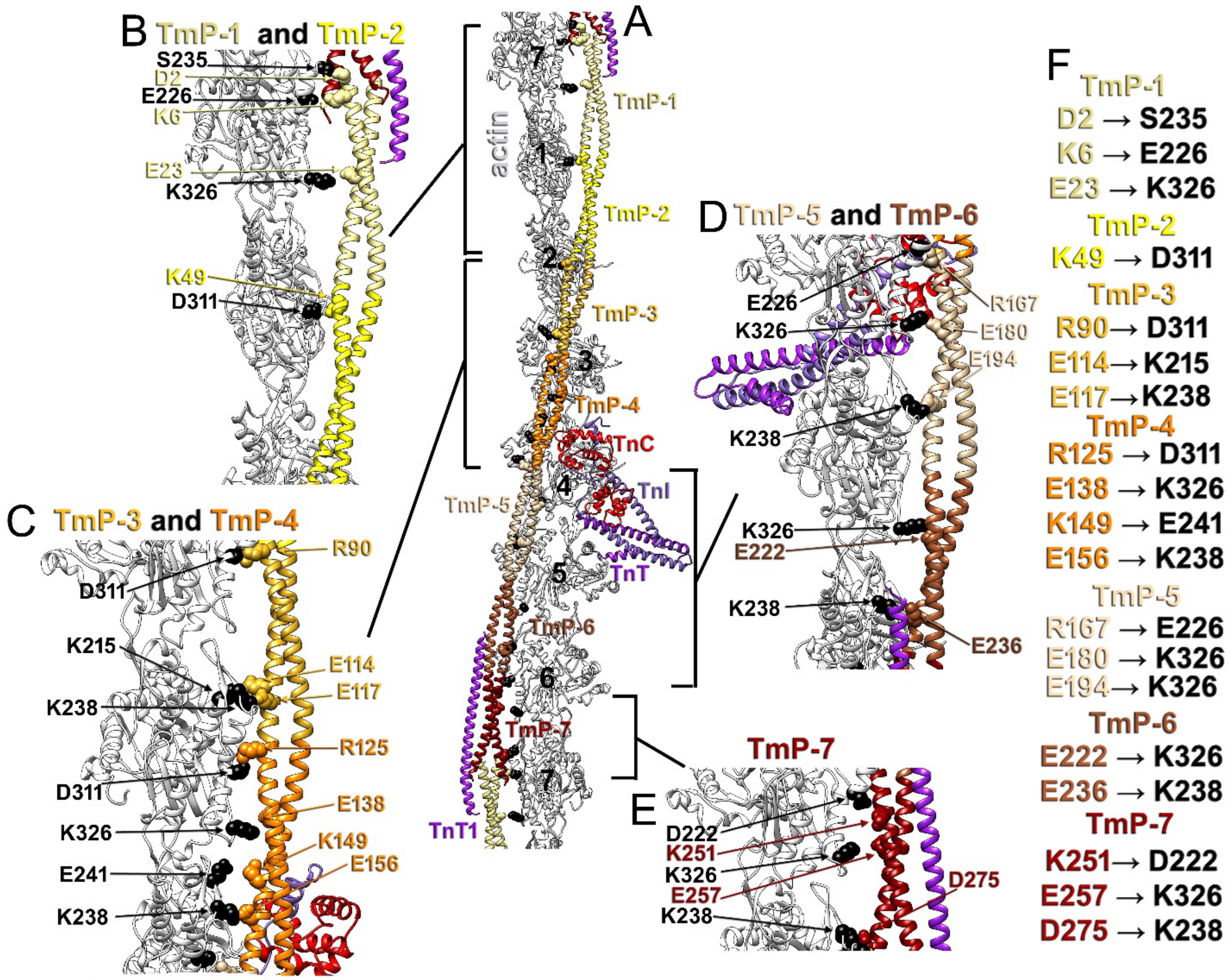

Figure S16. Interactions between actin and tropomyosin repeats. (**A** to **E**) Model of the cardiac TF using the same color code as in Fig. 1. Actin residues that interact with Tm are shown as black spheres, while Tm residues involved in actin binding are shown as spheres colored according to the Tm period. (**F**) Interactions between actin and Tm periods are arranged in a table.

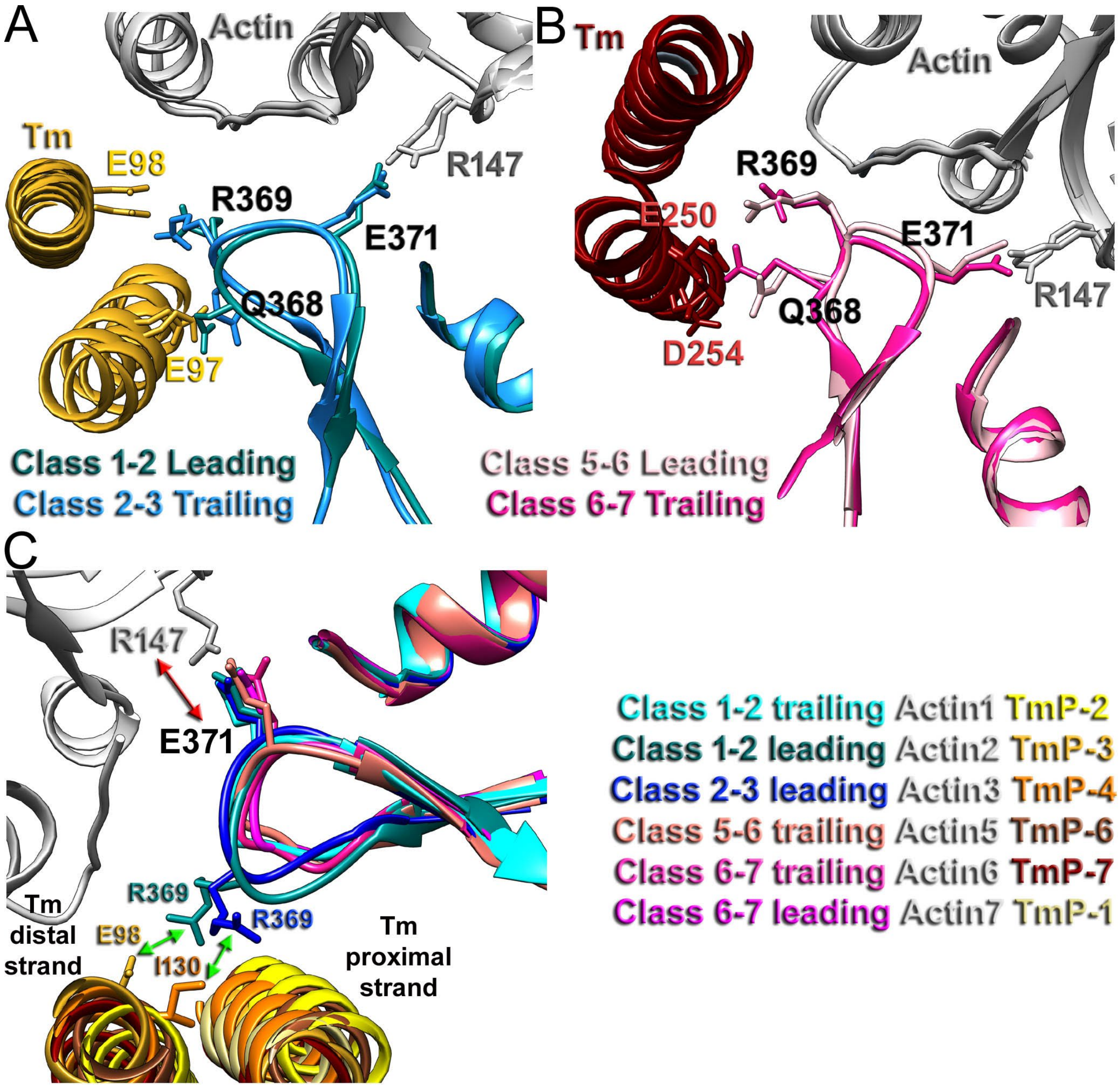

Figure S17. Interactions of myosin loop-4 with actin and Tm. (**A**) Loop-4 of the Class 1-2 leading head and loop-4 of the Class 2-3 trailing head form the same contacts with actin R147 (gray atoms) and Tm E97/E98 (gold atoms). (**B**) Loop-4 of the Class 5-6 leading head and loop-4 of the Class 6-7 trailing head form the same contacts with actin R147 (gray atoms) and Tm E250/D254 (dark red atoms). (**C**) The extended conformations of loop-4 from the Class 1-2 leading head and the Class 2-3 leading head correlate with their interactions with the far Tm strand (green arrows).

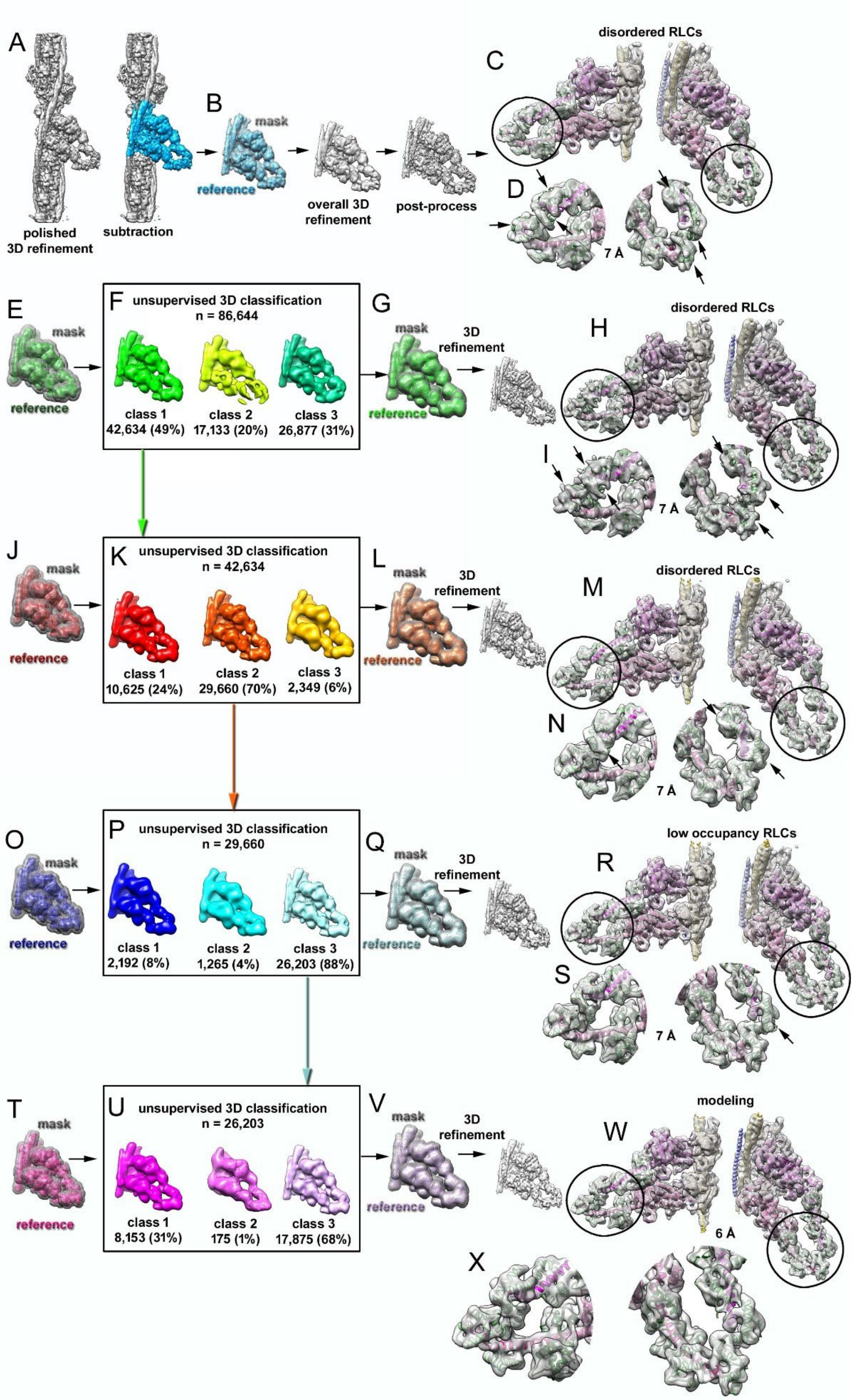
A
polished
3D refinement
subtraction
B
mask
reference
overall 3D
refinement
post-process
C
disordered RLCs
D
7 Å
E
mask
reference
F
unsupervised 3D classification
n = 86,644
class 1
42,634 (49%)
class 2
17,133 (20%)
class 3
26,877 (31%)
G
mask
reference
3D
refinement
H
disordered RLCs
I
7 Å
J
mask
reference
K
unsupervised 3D classification
n = 42,634
class 1
10,625 (24%)
class 2
29,660 (70%)
class 3
2,349 (6%)
L
mask
reference
3D
refinement
M
disordered RLCs
N
7 Å
O
mask
reference
P
unsupervised 3D classification
n = 29,660
class 1
2,192 (8%)
class 2
1,265 (4%)
class 3
26,203 (88%)
Q
mask
reference
3D
refinement
R
low occupancy RLCs
S
7 Å
T
mask
reference
U
unsupervised 3D classification
n = 26,203
class 1
8,153 (31%)
class 2
175 (1%)
class 3
17,875 (68%)
V
mask
reference
3D
refinement
W
modeling
6 Å
X

Figure S18. 3D classification and reconstruction algorithm for the RLCs of HMM bound to actin molecules 6 and 7. (**A** to **D**) Segmented densities of the HMM and actin molecules 6 and 7 were used for particle subtraction and then served as the reference (cerulean surface) and mask (gray mesh) for 3D refinement of those particles. The RLCs were not completely resolved (**C** to **D**, inserts and black arrows). (**E** to **H**) The map shown in (C) served as the reference and mask (green surface and gray mesh, respectively) for 3-class unsupervised sorting (**F**). The Class 1 3D class-average (green surface) was used as the initial reference and mask (green surface and gray mesh) for 3D refinement (**G**), which showed that the RLCs were not fully resolved (**H** to **I**; inserts and black arrows). (**J** to **N**) The resultant map from (H) served as the reference and mask (J, dark red surface and gray mesh, respectively) to reclassify the selected segments from the first round (n = 42,634) into 3 classes using an unsupervised approach (K). The Class 2 3D class average (dark orange surface) was used as the reference and mask (L, dark orange surface and gray mesh) for 3D refinement, indicating that the RLCs were not completely resolved despite a noticeable improvement (inserts and black arrows). (**O** to **R**) The map from (M) served as the reference, and the mask (O, navy blue surface and gray mesh), along with class 2 segments (n = 29,660), were used for the next round of unsupervised sorting (P). From this, the Class 3 3D class average (light blue surface) was used as the initial reference and mask (Q, light blue surface and gray mesh) for 3D refinement. 3D refinement showed that the trailing RLC was still not completely resolved (R-S, inserts and black arrows). (**T** to **X**) The map from (R) served as the reference and mask (T, deep pink surface and gray mesh, respectively) for the final round of the unsupervised sorting (U). (V) Class 3 (n=17,895) 3D class average (light pink surface) was used as an initial reference and mask (light pink surface and gray mesh) for 3D refinement. Both RLCs on the trailing and leading heads were resolved and clearly showed their secondary structure (W-X, inserts, and black arrows).

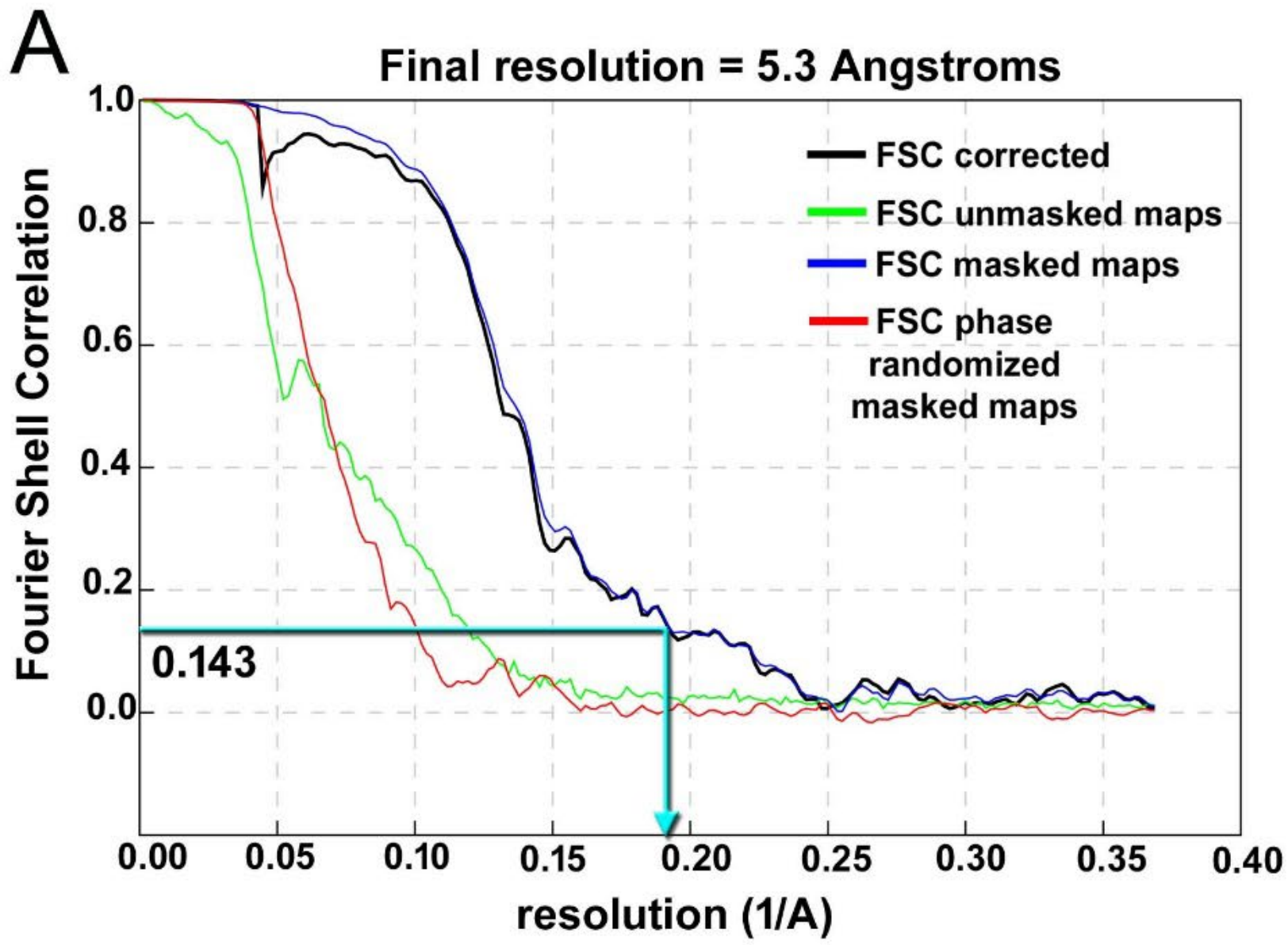

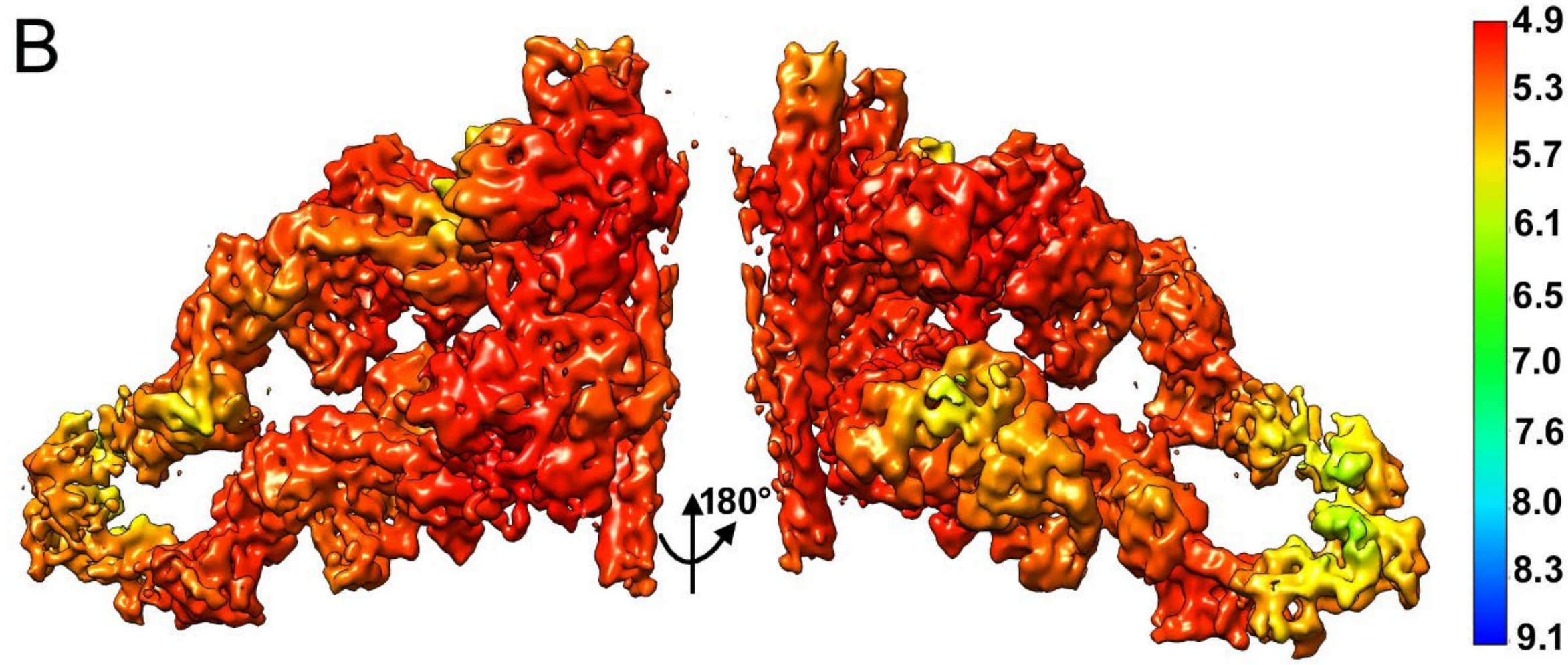

Figure S19. Resolution estimation for the Class 6-7 map with RLCs. (**A**) Fourier shell correlation (FSC) plot. The map's global resolution was calculated using an FSC criterion of 0.143. (**B**) Local resolution map determined by RELION.

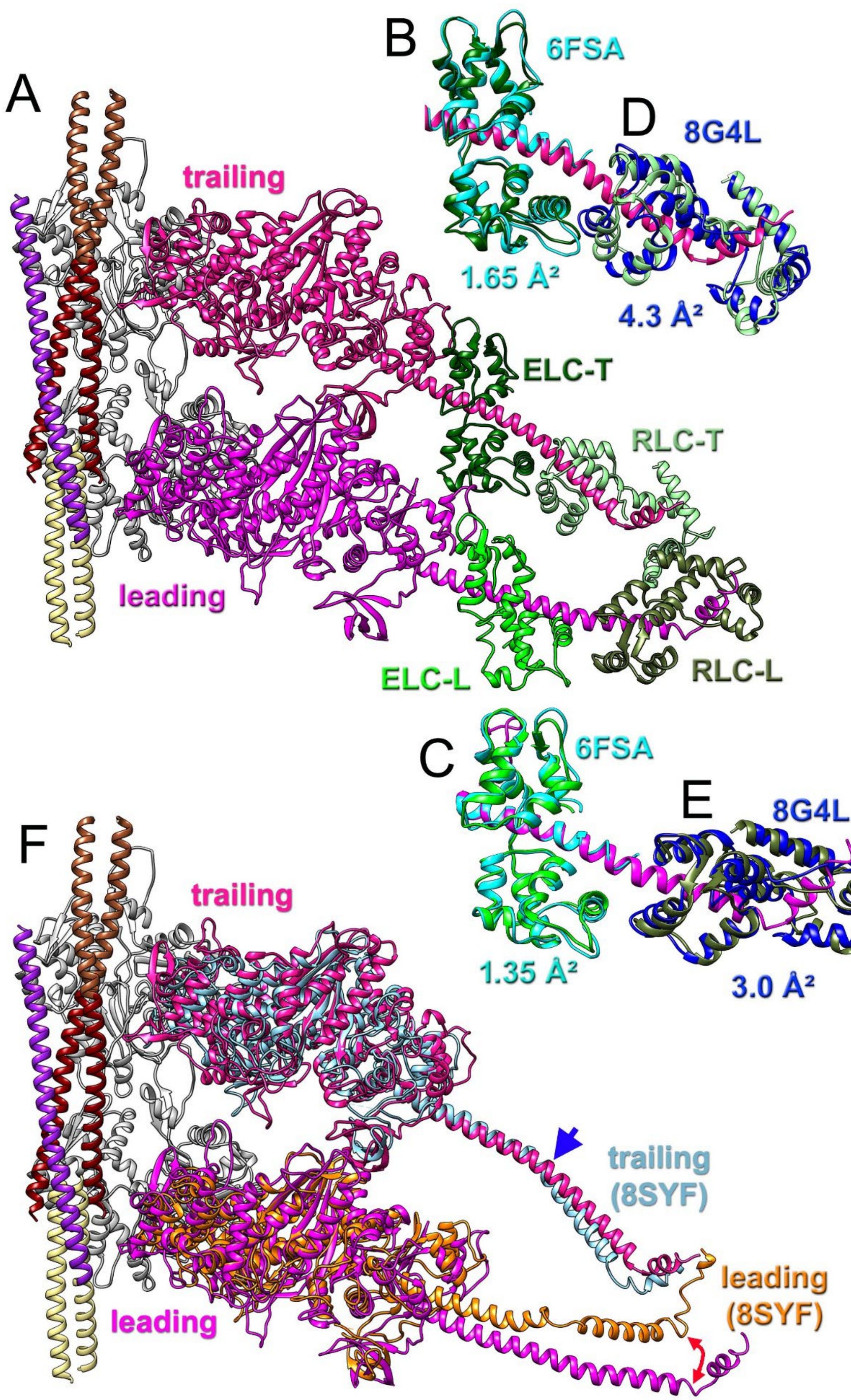

A
trailing
leading
ELC-T
RLC-T
ELC-L
RLC-L
B
6FSA
1.65 Å²
D
8G4L
4.3 Å²
C
6FSA
1.35 Å²
E
8G4L
3.0 Å²
F
trailing
leading
trailing (8SYF)
leading (8SYF)

Figure S20. Comparison of ELC and RLC atomic models and rod-domain structural states for trailing and leading heads obtained in the current study with previously published structures. (**A**) An overview of the atomic model of HMM bound to actins 6 and 7 that includes the neck region of myosin. The color codes are the same as in Fig. 1. (**B** to **E**) Alignment of previously published structures of ELC and RLC with RMSD values provided for every light chain. (B and C) Both ELCs in our atomic model exhibit excellent agreement with the high-resolution structure of cardiac myosin (PDB 6FSA) (*50*). (D and E) Both RLCs are consistent with the structure of the cardiac RLC reported previously by cryo-EM of the inhibited human cardiac myosin thick filament (PDB 8G4L) (*6*). (**F**) Structural alignment of the trailing (light blue) and leading (orange) heads from the cryo-EM structure of the HMM-ADP actomyosin complex (PDB 8SYF) (*30*) with our Class 6-7 structure, in which the trailing head rod domain is pink, and the leading head rod domain is magenta.

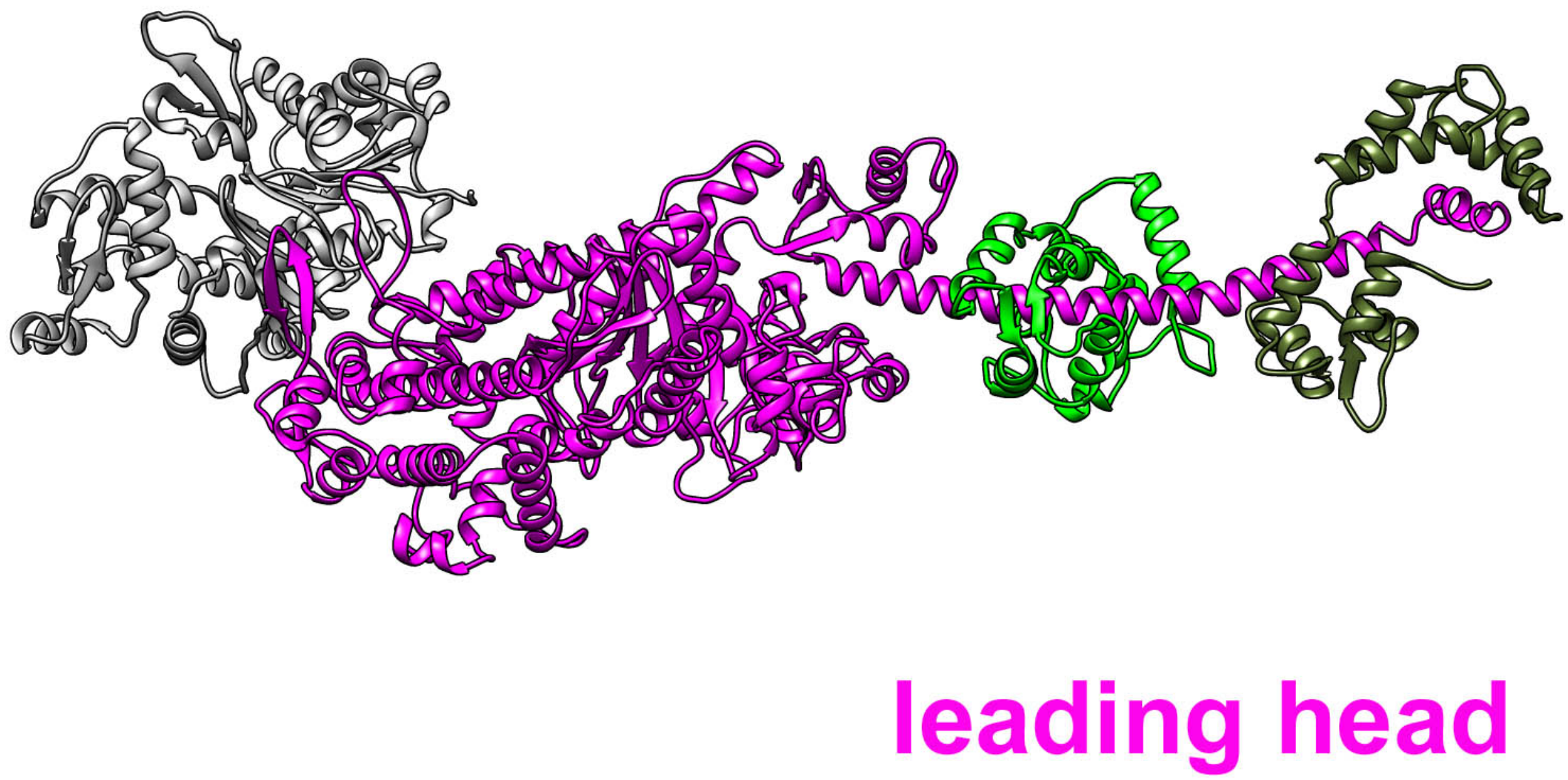

Movie S1. Transition of the myosin tail between its positions in the leading and trailing heads.

The color code for the myosin molecule is the same as in Fig. 1.

Table S1. Data collection and refinement statistics.

**Classes**	HMM bound to actins 6-7	HMM bound to actins 5-6	HMM bound to actins 2-3	HMM bound to actins 1-2	HMM bound to actins 6-7 with RLC
**Data collection**					
Magnification	65,000	65,000	65,000	65,000	65,000
Defocus range, µm	0.5 – 3.5	0.5 – 3.5	0.5 – 3.5	0.5 – 3.5	0.5 – 3.5
Voltage, kV	300	300	300	300	300
Microscope	Titan Krios	Titan Krios	Titan Krios	Titan Krios	Titan Krios
Camera	K3 (super-resolution mode)	K3 (super-resolution mode)	K3 (super-resolution mode)	K3 (super-resolution mode)	K3 (super-resolution mode)
Number of frames	40	40	40	40	40
Total electron dose, $e^-/Å^2$	34	34	34	34	34
Frames used for final reconstruction and dose used for final reconstruction, $e^-/Å^2$	Determined by Relion MotionCorr internal implementation	Determined by Relion MotionCorr internal implementation	Determined by Relion MotionCorr internal implementation	Determined by Relion MotionCorr internal implementation	Determined by Relion MotionCorr internal implementation
Pixel size, Å/px	0.678	0.678	0.678	0.678	0.678
**Particle statistics**					
Particles	86,644	55,229	31,180	54,451	17,875
Box size, Å	537	537	537	537	537
Pixel size, Å/px	1.356	1.356	1.356	1.356	1.356
**Resolution FSC 0.143, Å**	4.3 Å	4.6 Å	4.8 Å	4.5 Å	5.3 Å

**Resolution determined with Cryo-Res and local resolution maps used for modeling, Å**	3.8 Å	4.0 Å	4.2 Å	3.9 Å	6 Å

Table S2. Atomic models validation.

**Classes**	HMM bound to actins 6-7	HMM bound to actins 5-6	HMM bound to actins 2-3	HMM bound to actins 1-2	HMM bound to actins 6-7 with RLC
**Atoms**	30,836	33,975	33,217	30,685	26,682
**MolProbity score**	1.85	1.97	2.18	2.11	2.04
**Clash score**	6	6	7	7	8
**Ramachandran plot (%)**					
Outliers	0.00	0.07	0.15	0.13	0.00
Allowed	3.78	3.78	4.33	4.90	4.19
Favored	96.22	96.15	95.52	94.97	95.91
**Rotamer outliers (%)**	2.38	3.04	4.31	3.22	2.79
**Model vs Data correlation**	0.73	0.71	0.71	0.73	0.83
**Deposition ID**					
PDB	9YA8	9YAQ	9YJP	9YK9	9YKN
EMD	72724	72734	73030	73042	73055

Table S3. Statistics on the overall frequencies of structural classes (%)

Structural classes	Grids 1-3 (exp1)	Grids 4-6 (exp2)	Grids 7-10 (exp3)	Mean	SD
**Class1-2**	17.60	17.90	17.60	17.70	0.17
**Class2-3**	22.00	22.80	21.40	22.07	0.70
**Class5-6**	19.50	19.30	19.40	19.40	0.10
**Class6-7**	31.60	30.70	32.20	31.50	0.75
**Class7-1**	9.30	9.30	9.40	9.33	0.06

Table S4: Pairwise comparison of overall frequencies of structural classes.

Group 1	Group 2	Mean Difference	p-value	Statistical significance
Class1-2	Class2-3	+4.37	0.0010	Yes
Class1-2	Class5-6	+1.70	0.0088	Yes
Class1-2	Class6-7	+13.80	0.0010	Yes
Class1-2	Class7-1	-8.73	0.0010	Yes
Class2-3	Class5-6	-2.67	0.0010	Yes
Class2-3	Class6-7	+9.43	0.0010	Yes
Class2-3	Class7-1	-12.73	0.0010	Yes
Class5-6	Class6-7	+12.10	0.0010	Yes
Class5-6	Class7-1	-10.07	0.0010	Yes
Class6-7	Class7-1	-22.17	0.0010	Yes

Table S5: Structural class frequencies for upper and lower strands (%)

Structural classes	Upper strand Grids 1-3	Upper strand Grids 4-6	Upper strand Grids 7-10	Mean	SD	Lower Strand Grids 1-3	Lower Strand Grids 4-6	Lower Strand Grids 7-10	Mean	SD
class 1-2	17.1	17.6	17.1	17.30	0.29	18.1	18.2	18.1	18.10	0.06
class 2-3	21.7	22.2	20.5	21.50	0.87	22.4	23.3	22.3	22.70	0.55
class 5-6	18.1	18.3	18.9	18.40	0.42	20.8	20.3	19.8	20.30	0.50
class 6-7	32.7	31.7	32.9	32.40	0.64	30.5	29.7	31.5	30.60	0.90
class 7-1	10.4	10.2	10.6	10.40	0.20	8.2	8.5	8.3	8.30	0.15

Table S6: Pairwise Comparison Between Structural Modes of the Two Strands.

Upper strand	Lower strand	Mean Difference	p-value	Statistical significance
Class1-2	Class1-2	-0.8667	0.9421	No
Class2-3	Class2-3	-1.20	0.4318	No
Class5-6	Class5-6	-1.867	0.0167	Yes
Class6-7	Class6-7	1.867	0.0167	Yes
Class7-1	Class7-1	2.067	0.0058	Yes

Table S7. Missense cardiomyopathy-linked mutations related to the structure of the rigor cross-bridge.

ACTC1				
Residue; porcine sequence number	Mutation; human sequence number	Disease	Role and location	Reference
D24	D24N (D26N)	HCM	TnI binding	(*88*)
A26	A26V (A28V)	HCM	CM-loop binding	(*88*)
K50	K50T (K52T)	HCM	TnI binding	ClinVar https://www.ncbi.nlm.nih.gov/clinvar/RCV001961500/
Y91	Y91C/H (Y93C/H)	HCM/LVNC	TnI binding	(*89*, *90*)
R95	R95C (R97C)	HCM	Loop-2 binding	(*91*)
E99	E99K (E101K)	HCM	TnI binding	(*92*)
D222	D222Y (D224Y)	LVNC	TmP-7 binding	(*93*)
D311	D311H (D313H)	RCM	TmP-2, TmP-3, TmP-4 binding	(*94*)
MYH7				
R369	R369Q	Non-compaction CM	Tm binding	(*95*)
E374	E374V	HCM	Tm-P2 binding	(*88*)
E379	E379K	HCM	TnT binding	(*96*)
V404	V404L/M	HCM	Actin binding	(*97*), (*98*)
V406	V406M	HCM	Actin binding	(*99*)
V411	V411I	HCM	Actin binding	(*100*)

E536	E536D	HCM	Actin binding	(*101*)
F540	F540L	DCM	Actin binding	(*102*)
K639	K639E	LVNC	Actin binding	(*103*)
R723	R723C/G/H	HCM	ELC binding	(*104*), (*105*), ClinVar https://www.ncbi.nlm.nih.gov/clinvar/RCV000148962/
R787	R787C/H	HCM	ELC binding	(*91*), (*106*)
R793	R793Q	HCM	ELC binding	(*107*)
R807	R807G/H	LVNC/HCM	RLC binding	(*93*), (*89*)
R819	R819Q/W	HCM/DCM	RLC binding	(*96*), (*88*)
K835	K835T	HCM	RLC binding	(*108*)
K837	K837M/R		RLC binding	ClinVar https://www.ncbi.nlm.nih.gov/clinvar/RCV000467403/ https://www.ncbi.nlm.nih.gov/clinvar/RCV001808158/
		MYL3		
E153	E152K	HCM	Myosin converter interaction	(*109*)
		MYL2		
E22	E22K	HCM	RLC-RLC interaction	(*110*)
R58	R58Q/L	HCM	RLC-RLC interaction	(*111*), ClinVar https://www.ncbi.nlm.nih.gov/clinvar/RCV000639677/
E163	E163A	HCM	Myosin rod interaction	ClinVar https://www.ncbi.nlm.nih.gov/clinvar/RCV000036409/
D166	D166V	HCM	Myosin rod interaction	(*106*)
		TPM1		

E16	E16Q	HCM	Actin binding	(*88*)
D20	D20N	HCM	Actin binding	(88)
E23	E23G	DCM	Actin binding	(*112*)
E54	E54K	DCM	Loop-4 binding	(*113*)
D55	D55N/Y	DCM	Loop-4 binding	(*114*), ClinVar https://www.ncbi.nlm.nih.gov/clinvar/RCV000852460/
D58	D58H	HCM	Loop-4 binding	(*115*)
E96	E96G	HCM	Loop-4 binding	ClinVar https://www.ncbi.nlm.nih.gov/clinvar/RCV001996289/
E98	E98K	HCM	Loop-4 binding	(*116*)
E114	E114G/Q	DCM	Actin binding	(*114*), (*117*)
I130	I130V/T	HCM/ congenital heart defect	Loop-4 binding	(*118*), ClinVar https://www.ncbi.nlm.nih.gov/clinvar/RCV000549273/
E180	E180G/V/K	HCM	Actin binding	(*119*), (*120*), ClinVar https://www.ncbi.nlm.nih.gov/clinvar/RCV001298950/
S215	S215L	HCM	Loop-4 binding	(*91*)
E250	E250V	CM	Loop-4 binding	ClinVar https://www.ncbi.nlm.nih.gov/clinvar/RCV000852462/
E254	E254G	HCM	Loop-4 binding	(88)
TNNI3				
K37	K36Q	DCM	TnC interaction	(*121*)
D128	D127Y	RCM/HCM	Actin binding	(*122*)
R147	R146S	HCM	Actin binding	ClinVar https://www.ncbi.nlm.nih.gov/clinvar/RCV001256923/

TNNT2				
R91	R92L/Q/W	HCM	Loop-4 binding	(*123*), (*119*), (*124*), (*125*)
R202	R205L/W	DCM	Actin binding	(*126*), (*127*)
N266	N269D/K	HCM	Loop-2 binding	(*88*) (*101*)

REFERENCES

1. I. Pepper, V. E. Galkin, Actomyosin complex. *Subcell. Biochem.* **99**, 421–470 (2022).

2. M. A. Hartman, D. Finan, S. Sivaramakrishnan, J. A. Spudich, Principles of unconventional myosin function and targeting. *Annu. Rev. Cell Dev. Biol.* **27**, 133–155 (2011).

3. H. L. Sweeney, S. S. Rosenfeld, F. Brown, L. Faust, J. Smith, J. Xing, L. A. Stein, J. R. Sellers, Kinetic tuning of myosin via a flexible loop adjacent to the nucleotide binding pocket. *J. Biol. Chem.* **273**, 6262–6270 (1998).

4. H. Shuman, M. J. Greenberg, A. Zwolak, T. Lin, C. V. Sindelar, R. Dominguez, E. M. Ostap, A vertebrate myosin-I structure reveals unique insights into myosin mechanochemical tuning. *Proc. Natl. Acad. Sci. U.S.A.* **111**, 2116–2121 (2014).

5. A. D. McLachlan, M. Stewart, Tropomyosin coiled-coil interactions: Evidence for an unstaggered structure. *J. Mol. Biol.* **98**, 293–304 (1975).

6. D. Dutta, V. Nguyen, K. S. Campbell, R. Padron, R. Craig, Cryo-EM structure of the human cardiac myosin filament. *Nature* **623**, 853–862 (2023).

7. J. R. Sellers, Myosins: A diverse superfamily. *Biochim. Biophys. Acta* **1496**, 3–22 (2000).

8. S. Lowey, H. S. Slayter, A. G. Weeds, H. Baker, Substructure of the myosin molecule. I. Subfragments of myosin by enzymic degradation. *J. Mol. Biol.* **42**, 1–29 (1969).

9. A. Houdusse, H. L. Sweeney, How myosin generates force on actin filaments. *Trends Biochem. Sci.* **41**, 989–997 (2016).

10. A. J. Fisher, C. A. Smith, J. Thoden, R. Smith, K. Sutoh, H. M. Holden, I. Rayment, Structural studies of myosin:nucleotide complexes: A revised model for the molecular basis of muscle contraction. *Biophys. J.* **68**, 19S–26S (1995).

11. A. Houdusse, A. G. Szent-Gyorgyi, C. Cohen, Three conformational states of scallop myosin S1. *Proc. Natl. Acad. Sci. U.S.A.* **97**, 11238–11243 (2000).

12. C. Risi, L. U. Schafer, B. Belknap, I. Pepper, H. D. White, G. F. Schroder, V. E. Galkin, High-resolution Cryo-EM structure of the cardiac actomyosin complex. *Structure* **29**, 50–60. e4 (2021).

13. M. H. Doran, M. J. Rynkiewicz, D. Rasicci, S. M. L. Bodt, M. E. Barry, E. Bullitt, C. M. Yengo, J. R. Moore, W. Lehman, Conformational changes linked to ADP release from human cardiac myosin bound to actin-tropomyosin. *J. Gen. Physiol.* **155**, e202213267 (2023).

14. S. Pospich, H. L. Sweeney, A. Houdusse, S. Raunser, High-resolution structures of the actomyosin-V complex in three nucleotide states provide insights into the force generation mechanism. *eLife* **10**, e73724 (2021).

15. T. Fujii, K. Namba, Structure of actomyosin rigour complex at 5.2 Å resolution and insights into the ATPase cycle mechanism. *Nat. Commun.* **8**, 13969 (2017).

16. J. von der Ecken, S. M. Heissler, S. Pathan-Chhatbar, D. J. Manstein, S. Raunser, Cryo-EM structure of a human cytoplasmic actomyosin complex at near-atomic resolution. *Nature* **534**, 724–728 (2016).

17. P. S. Gurel, L. Y. Kim, P. V. Ruijgrok, T. Omabegho, Z. Bryant, G. M. Alushin, Cryo-EM structures reveal specialization at the myosin VI-actin interface and a mechanism of force sensitivity. *eLife* **6**, e31125 (2017).

18. A. Mentes, A. Huehn, X. Liu, A. Zwolak, R. Dominguez, H. Shuman, E. M. Ostap, C. V. Sindelar, High-resolution cryo-EM structures of actin-bound myosin states reveal the mechanism of myosin force sensing. *Proc. Natl. Acad. Sci. U.S.A.* **115**, 1292–1297 (2018).

19. D. P. Klebl, S. N. McMillan, C. Risi, E. Forgacs, B. Virok, J. L. Atherton, S. A. Harris, M. Stofella, D. A. Winkelmann, F. Sobott, V. E. Galkin, P. J. Knight, S. P. Muench, C. A. Scarff, H. D. White, Swinging lever mechanism of myosin directly shown by time-resolved cryo-EM. *Nature* **642**, 519–526 (2025).

20. Y. Y. Toyoshima, S. J. Kron, E. M. McNally, K. R. Niebling, C. Toyoshima, J. A. Spudich, Myosin subfragment-1 is sufficient to move actin filaments in vitro. *Nature* **328**, 536–539 (1987).

21. D. Tamborrini, Z. Wang, T. Wagner, S. Tacke, M. Stabrin, M. Grange, A. L. Kho, M. Rees, P. Bennett, M. Gautel, S. Raunser, Structure of the native myosin filament in the relaxed cardiac sarcomere. *Nature* **623**, 863–871 (2023).

22. Tyska, D. E. Dupuis, W. H. Guilford, J. B. Patlak, G. S. Waller, K. M. Trybus, D. M. Warshaw, S. Lowey, Two heads of myosin are better than one for generating force and motion. *Proc. Natl. Acad. Sci. U.S.A.* **96**, 4402–4407 (1999).

23. P. B. Conibear, M. A. Geeves, Cooperativity between the two heads of rabbit skeletal muscle heavy meromyosin in binding to actin. *Biophys. J.* **75**, 926–937 (1998).

24. E. Brunello, M. Reconditi, R. Elangovan, M. Linari, Y. B. Sun, T. Narayanan, P. Panine, G. Piazzesi, M. Irving, V. Lombardi, Skeletal muscle resists stretch by rapid binding of the second motor domain of myosin to actin. *Proc. Natl. Acad. Sci. U.S.A.* **104**, 20114–20119 (2007).

25. Z. Wang, M. Grange, T. Wagner, A. L. Kho, M. Gautel, S. Raunser, The molecular basis for sarcomere organization in vertebrate skeletal muscle. *Cell* **184**, 2135–2150.e13 (2021).

26. Z. Wang, M. Grange, S. Pospich, T. Wagner, A. L. Kho, M. Gautel, S. Raunser, Structures from intact myofibrils reveal mechanism of thin filament regulation through nebulin. *Science* **375**, eabn1934 (2022).

27. I. Rayment, H. M. Holden, M. Whittaker, C. B. Yohn, M. Lorenz, K. C. Holmes, R. A. Milligan, Structure of the actin-myosin complex and its implications for muscle contraction. *Science* **261**, 58–65 (1993).

28. I. Rayment, W. R. Rypniewski, K. Schmidt-Bäse, R. Smith, D. R. Tomchick, M. M. Benning, D. A. Winkelmann, G. Wesenberg, H. M. Holden, Three-dimensional structure of myosin subfragment-1: A molecular motor. *Science* **261**, 50–58 (1993).

29. M. H. Doran, E. Pavadai, M. J. Rynkiewicz, J. Walklate, E. Bullitt, J. R. Moore, M. Regnier, M. A. Geeves, W. Lehman, Cryo-EM and molecular docking shows myosin loop 4 contacts actin and tropomyosin on thin filaments. *Biophys. J.* **119**, 821–830 (2020).

30. A. Hojjatian, D. W. Taylor, N. Daneshparvar, P. M. Fagnant, K. M. Trybus, K. A. Taylor, Double-headed binding of myosin II to F-actin shows the effect of strain on head structure. *J. Struct. Biol.* **215**, 107995 (2023).

31. D. F. A. McKillop, M. A. Geeves, Regulation of the interaction between actin and myosin subfragment-1: Evidence for three states of the thin filament. *Biophys. J.* **65**, 693–701 (1993).

32. S. Ebashi, Calcium ions and muscle contraction. *Nature* **240**, 217–218 (1972).

33. W. Drabikowski, D. R. Kominz, K. Maruyama, Effect of troponin on the reversibility of tropomyosin binding to F-actin. *J. Biochem.* **63**, 802–804 (1968).

34. S. S. Lehrer, E. P. Morris, Dual effects of tropomyosin and troponin-tropomyosin on actomyosin subfragment 1 ATPase. *J. Biol. Chem.* **257**, 8073–8080 (1982).

35. D. A. Martyn, M. Regnier, D. Xu, A. M. Gordon, Ca^{2+} - and cross-bridge-dependent changes in *N*- and *C*-terminal structure of troponin C in rat cardiac muscle. *Biophys. J.* **80**, 360–370 (2001).

36. G. N. Phillips Jr., Construction of an atomic model for tropomyosin and implications for interactions with actin. *J. Mol. Biol.* **192**, 128–131 (1986).

37. B. Barua, M. C. Pamula, S. E. Hitchcock-DeGregori, Evolutionarily conserved surface residues constitute actin binding sites of tropomyosin. *Proc. Natl. Acad. Sci. U.S.A.* **108**, 10150–10155 (2011).

38. N. J. Greenfield, Y. J. Huang, G. V. T. Swapna, A. Bhattacharya, B. Rapp, A. Singh, G. T. Montelione, S. E. Hitchcock-DeGregori, Solution NMR structure of the junction between tropomyosin molecules: Implications for actin binding and regulation. *J. Mol. Biol.* **364**, 80–96 (2006).

39. C. M. Risi, B. Belknap, H. D. White, K. Dryden, J. R. Pinto, P. B. Chase, V. E. Galkin, High-resolution cryo-EM structure of the junction region of the native cardiac thin filament in relaxed state. *PNAS Nexus* **2**, pgac298 (2023).

40. Y. Yamada, K. Namba, T. Fujii, Cardiac muscle thin filament structures reveal calcium regulatory mechanism. *Nat. Commun.* **11**, 153 (2020).

41. C. M. Risi, I. Pepper, B. Belknap, M. Landim-Vieira, H. D. White, K. Dryden, J. R. Pinto, P. B. Chase, V. E. Galkin, The structure of the native cardiac thin filament at systolic Ca^{2+} levels. *Proc. Natl. Acad. Sci. U.S.A.* **118**, e2024288118 (2021).

42. C. M. Risi, M. Landim-Vieira, B. Belknap, P. B. Chase, J. R. Pinto, V. E. Galkin, The role of the troponin T interactions with actin in regulation of cardiac thin filament revealed by the troponin T pathogenic variant Ile79Asn. *J. Mol. Cell. Cardiol.* **204**, 55–67 (2025).

43. C. M. Risi, B. Belknap, J. Atherton, I. L. Coscarella, H. D. White, P. Bryant Chase, J. R. Pinto, V. E. Galkin, Troponin structural dynamics in the native cardiac thin filament revealed by cryo electron microscopy. *J. Mol. Biol.* **436**, 168498 (2024).

44. M. A. Geeves, S. S. Lehrer, Dynamics of the muscle thin filament regulatory switch: The size of the cooperative unit. *Biophys. J.* **67**, 273–282 (1994).

45. R. Desai, M. A. Geeves, N. M. Kad, Using fluorescent myosin to directly visualize cooperative activation of thin filaments. *J. Biol. Chem.* **290**, 1915–1925 (2015).

46. S. Subramaniam, L. A. Earl, V. Falconieri, J. L. Milne, E. H. Egelman, Resolution advances in cryo-EM enable application to drug discovery. *Curr. Opin. Struct. Biol.* **41**, 194–202 (2016).

47. S. H. W. Scheres, RELION: Implementation of a Bayesian approach to cryo-EM structure determination. *J. Struct. Biol.* **180**, 519–530 (2012).

48. M. Dai, Z. Dong, K. Xu, Q. C. Zhang, CryoRes: Local resolution estimation of cryo-EM density maps by deep learning. *J. Mol. Biol.* **435**, 168059 (2023).

49. J. von der Ecken, M. Muller, W. Lehman, D. J. Manstein, P. A. Penczek, S. Raunser, Structure of the F-actin-tropomyosin complex. *Nature* **519**, 114–117 (2015).

50. J. Robert-Paganin, D. Auguin, A. Houdusse, Hypertrophic cardiomyopathy disease results from disparate impairments of cardiac myosin function and auto-inhibition. *Nat. Commun.* **9**, 4019 (2018).

51. A. Grinzato, D. Auguin, C. Kikuti, N. Nandwani, D. Moussaoui, D. Pathak, E. Kandiah, K. M. Ruppel, J. A. Spudich, A. Houdusse, J. Robert-Paganin, Cryo-EM structure of the folded-back state of human β-cardiac myosin. *Nat. Commun.* **14**, 3166 (2023).

52. M. X. Li, L. Spyracopoulos, B. D. Sykes, Binding of cardiac troponin-$I_{147\text{-}163}$ induces a structural opening in human cardiac troponin-C. *Biochemistry* **38**, 8289–8298 (1999).

53. Y. Shi, L. A. Blackwell, R. K. Schroy, B. M. Cleland, C. M. Risi, M. S. Parvatiyar, J. R. Pinto, V. E. Galkin, P. B. Chase, A central role for troponin C amino-terminal α-helix in vertebrate thin filament Ca-activation. *Physchem* **5**, 16 (2025).

54. R. L. Anderson, D. V. Trivedi, S. S. Sarkar, M. Henze, W. Ma, H. Gong, C. S. Rogers, J. M. Gorham, F. L. Wong, M. M. Morck, J. G. Seidman, K. M. Ruppel, T. C. Irving, R. Cooke, E. M. Green, J. A. Spudich, Deciphering the super relaxed state of human β-cardiac myosin and the mode of action of mavacamten from myosin molecules to muscle fibers. *Proc. Natl. Acad. Sci. U.S.A.* **115**, E8143–E8152 (2018).

55. N. Albet-Torres, M. J. Bloemink, T. Barman, R. Candau, K. Frölander, M. A. Geeves, K. Golker, C. Herrmann, C. Lionne, C. Piperio, S. Schmitz, C. Veigel, A. Månsson, Drug effect unveils inter-head cooperativity and strain-dependent ADP release in fast skeletal actomyosin. *J. Biol. Chem.* **284**, 22926–22937 (2009).

56. A. Månsson, Actomyosin-ADP states, interhead cooperativity, and the force-velocity relation of skeletal muscle. *Biophys. J.* **98**, 1237–1246 (2010).

57. C. Liu, M. Kawana, D. Song, K. M. Ruppel, J. A. Spudich, Controlling load-dependent kinetics of β-cardiac myosin at the single-molecule level. *Nat. Struct. Mol. Biol.* **25**, 505–514 (2018).

58. H. Onishi, S. V. Mikhailenko, M. F. Morales, Toward understanding actin activation of myosin ATPase: The role of myosin surface loops. *Proc. Natl. Acad. Sci. U.S.A.* **103**, 6136–6141 (2006).

59. K. Ito, Y. Yamaguchi, K. Yanase, Y. Ichikawa, K. Yamamoto, Unique charge distribution in surface loops confers high velocity on the fast motor protein Chara myosin. *Proc. Natl. Acad. Sci. U.S.A.* **106**, 21585–21590 (2009).

60. J. Robert-Paganin, O. Pylypenko, C. Kikuti, H. L. Sweeney, A. Houdusse, Force generation by myosin motors: A structural perspective. *Chem. Rev.* **120**, 5–35 (2020).

61. R. D. Bremel, A. Weber, Cooperation within actin filament in vertebrate skeletal muscle. *Nat. New Biol.* **238**, 97–101 (1972).

62. A. S. Zot, J. D. Potter, Reciprocal coupling between troponin C and myosin crossbridge attachment. *Biochemistry* **28**, 6751–6756 (1989).

63. Y. Wang, K. Ajtai, K. Kazmierczak, D. Szczesna-Cordary, T. P. Burghardt, N-terminus of cardiac myosin essential light chain modulates myosin step-size. *Biochemistry* **55**, 186–198 (2016).

64. K. Kazmierczak, Y. Xu, M. Jones, G. Guzman, O. M. Hernandez, W. G. L. Kerrick, D. Szczesna-Cordary, The role of the N-terminus of the myosin essential light chain in cardiac muscle contraction. *J. Mol. Biol.* **387**, 706–725 (2009).

65. M. S. Miller, B. M. Palmer, S. Ruch, L. A. Martin, G. P. Farman, Y. Wang, J. Robbins, T. C. Irving, D. W. Maughan, The essential light chain N-terminal extension alters force and fiber kinetics in mouse cardiac muscle. *J. Biol. Chem.* **280**, 34427–34434 (2005).

66. P. Muthu, L. Wang, C. C. Yuan, K. Kazmierczak, W. Huang, O. M. Hernandez, M. Kawai, T. C. Irving, D. Szczesna-Cordary, Structural and functional aspects of the myosin essential light chain in cardiac muscle contraction. *FASEB J.* **25**, 4394–4405 (2011).

67. K. Pant, J. Watt, M. Greenberg, M. Jones, D. Szczesna-Cordary, J. R. Moore, Removal of the cardiac myosin regulatory light chain increases isometric force production. *FASEB J.* **23**, 3571–3580 (2009).

68. D. R. Jackson Jr., J. E. Baker, The energetics of allosteric regulation of ADP release from myosin heads. *Phys. Chem. Chem. Phys.* **11**, 4808–4814 (2009).

69. M. Kovács, K. Thirumurugan, P. J. Knight, J. R. Sellers, Load-dependent mechanism of nonmuscle myosin 2. *Proc. Natl. Acad. Sci. U.S.A.* **104**, 9994–9999 (2007).

70. R. F. Siemankowski, P. Dreizen, Canine cardiac myosin with special reference to pressure overload cardiac hypertrophy. II. Myosin ATPase. II. Myosin ATPase. *J. Biol. Chem.* **253**, 8659–8665 (1978).

71. R. F. Siemankowski, H. D. White, Kinetics of the interaction between actin, ADP, and cardiac myosin-S1. *J. Biol. Chem.* **259**, 5045–5053 (1984).

72. Y. Liu, H. D. White, B. Belknap, D. A. Winkelmann, E. Forgacs, Omecamtiv Mecarbil modulates the kinetic and motile properties of porcine β-Cardiac myosin. *Biochemistry* **54**, 1963–1975 (2015).

73. E. F. Pettersen, T. D. Goddard, C. C. Huang, G. S. Couch, D. M. Greenblatt, E. C. Meng, T. E. Ferrin, UCSF Chimera—A visualization system for exploratory research and analysis. *J. Comput. Chem.* **25**, 1605–1612 (2004).

74. R. T. Kidmose, J. Juhl, P. Nissen, T. Boesen, J. L. Karlsen, B. P. Pedersen, Namdinator - automatic molecular dynamics flexible fitting of structural models into cryo-EM and crystallography experimental maps. *IUCrJ* **6**, 526–531 (2019).

75. T. I. Croll, ISOLDE: A physically realistic environment for model building into low-resolution electron-density maps. *Acta Crystallogr. D Struct. Biol.* **74**, 519–530 (2018).

76. D. Liebschner, P. V. Afonine, M. L. Baker, G. Bunkóczi, V. B. Chen, T. I. Croll, B. Hintze, L. W. Hung, S. Jain, A. J. McCoy, N. W. Moriarty, R. D. Oeffner, B. K. Poon, M. G. Prisant, R. J. Read, J. S. Richardson, D. C. Richardson, M. D. Sammito, O. V. Sobolev, D. H. Stockwell, T. C. Terwilliger, A. G. Urzhumtsev, L. L. Videau, C. J. Williams, P. D. Adams, Macromolecular structure determination using X-rays, neutrons and electrons: Recent developments in Phenix. *Acta Crystallogr. D Struct. Biol.* **75**, 861–877 (2019).

77. A. Rohou, N. Grigorieff, CTFFIND4: Fast and accurate defocus estimation from electron micrographs. *J. Struct. Biol.* **192**, 216–221 (2015).

78. A. E. Leschziner, E. Nogales, Visualizing flexibility at molecular resolution: Analysis of heterogeneity in single-particle electron microscopy reconstructions. *Annu. Rev. Biophys. Biomol. Struct.* **36**, 43–62 (2007).

79. R. A. Fisher, *Statistical methods for research workers* (Oliver and Boyd, Edinburgh, London, 1925), pp. 66–70.

80. J. W. Tukey, Comparing individual means in the analysis of variance. *Biometrics* **5**, 99–114 (1949).

81. S. Holm, A simple sequentially rejective multiple test procedure. *Scand. J. Stat.* **6**, 65–70 (1979).

82. G. D. Pintilie, J. Zhang, T. D. Goddard, W. Chiu, D. C. Gossard, Quantitative analysis of cryo-EM density map segmentation by watershed and scale-space filtering, and fitting of structures by alignment to regions. *J. Struct. Biol.* **170**, 427–438 (2010).

83. E. C. Meng, T. D. Goddard, E. F. Pettersen, G. S. Couch, Z. J. Pearson, J. H. Morris, T. E. Ferrin, UCSF ChimeraX: Tools for structure building and analysis. *Protein Sci.* **32**, e4792 (2023).

84. E. F. Pettersen, T. D. Goddard, C. C. Huang, E. C. Meng, G. S. Couch, T. I. Croll, J. H. Morris, T. E. Ferrin, UCSF ChimeraX: Structure visualization for researchers, educators, and developers. *Protein Sci.* **30**, 70–82 (2021).

85. J. Jumper, R. Evans, A. Pritzel, T. Green, M. Figurnov, O. Ronneberger, K. Tunyasuvunakool, R. Bates, A. Žídek, A. Potapenko, A. Bridgland, C. Meyer, S. A. A. Kohl, A. J. Ballard, A. Cowie, B. Romera-Paredes, S. Nikolov, R. Jain, J. Adler, T. Back, S. Petersen, D. Reiman, E. Clancy, M. Zielinski, M. Steinegger, M. Pacholska, T. Berghammer, S. Bodenstein, D. Silver, O. Vinyals, A. W. Senior, K. Kavukcuoglu, P. Kohli, D. Hassabis, Highly accurate protein structure prediction with AlphaFold. *Nature* **596**, 583–589 (2021).

86. M. Varadi, D. Bertoni, P. Magana, U. Paramval, I. Pidruchna, M. Radhakrishnan, M. Tsenkov, S. Nair, M. Mirdita, J. Yeo, O. Kovalevskiy, K. Tunyasuvunakool, A. Laydon, A. Žídek, H. Tomlinson, D. Hariharan, J. Abrahamson, T. Green, J. Jumper, E. Birney, M. Steinegger, D. Hassabis, S. Velankar, AlphaFold Protein Structure Database in 2024: Providing structure coverage for over 214 million protein sequences. *Nucleic Acids Res.* **52**, D368–D375 (2024).

87. A. Waterhouse, M. Bertoni, S. Bienert, G. Studer, G. Tauriello, R. Gumienny, F. T. Heer, T. A. P. de Beer, C. Rempfer, L. Bordoli, R. Lepore, T. Schwede, SWISS-MODEL: Homology modelling of protein structures and complexes. *Nucleic Acids Res.* **46**, W296–W303 (2018).

88. R. Walsh, K. L. Thomson, J. S. Ware, B. H. Funke, J. Woodley, K. J. McGuire, F. Mazzarotto, E. Blair, A. Seller, J. C. Taylor, E. V. Minikel, Exome Aggregation Consortium, D. G. MacArthur, M. Farrall, S. A. Cook, H. Watkins, Reassessment of Mendelian gene pathogenicity using 7,855 cardiomyopathy cases and 60,706 reference samples. *Genet. Med.* **19**, 192–203 (2017).

89. C. Burns, R. D. Bagnall, L. Lam, C. Semsarian, J. Ingles, Multiple gene variants in hypertrophic cardiomyopathy in the era of next-generation sequencing. *Circ. Cardiovasc. Genet.* **10**, e001666 (2017).

90. C. Wang, Y. Hata, K. Hirono, A. Takasaki, S. W. Ozawa, H. Nakaoka, K. Saito, N. Miyao, M. Okabe, K. Ibuki, N. Nishida, H. Origasa, X. Yu, N. E. Bowles, F. Ichida, for LVNC Study Collaborators, A wide and specific spectrum of genetic variants and genotype-phenotype correlations revealed by next-generation sequencing in patients with left ventricular noncompaction. *J. Am. Heart Assoc.* **6**, e006210 (2017).

91. H. Morita, H. L. Rehm, A. Menesses, B. McDonough, A. E. Roberts, R. Kucherlapati, J. A. Towbin, J. G. Seidman, C. E. Seidman, Shared genetic causes of cardiac hypertrophy in children and adults. *N. Engl. J. Med.* **358**, 1899–1908 (2008).

92. T. M. Olson, T. P. Doan, N. Y. Kishimoto, F. G. Whitby, M. J. Ackerman, L. Fananapazir, Inherited and de novo mutations in the cardiac actin gene cause hypertrophic cardiomyopathy. *J. Mol. Cell. Cardiol.* **32**, 1687–1694 (2000).

93. P. Richard, F. Ader, M. Roux, E. Donal, J.-C. Eicher, N. Aoutil, O. Huttin, C. Selton-Suty, D. Coisne, G. Jondeau, T. Damy, N. Mansencal, A.-C. Casalta, N. Michel, J. Haentjens, L. Faivre, C. Lavoute, K. Nguyen, D.-A. Tregouet, G. Habib, P. Charron, Targeted panel sequencing in adult patients with left ventricular non-compaction reveals a large genetic heterogeneity. *Clin. Genet.* **95**, 356–367 (2019).

94. J. P. Kaski, P. Syrris, M. Burch, M. T. Tomé-Esteban, M. Fenton, M. Christiansen, P. S. Andersen, N. Sebire, M. Ashworth, J. E. Deanfield, W. J. McKenna, P. M. Elliott, Idiopathic restrictive cardiomyopathy in children is caused by mutations in cardiac sarcomere protein genes. *Heart* **94**, 1478–1484 (2008).

95. L. M. Dellefave, P. Pytel, S. Mewborn, B. Mora, D. L. Guris, S. Fedson, D. Waggoner, I. Moskowitz, E. M. McNally, Sarcomere mutations in cardiomyopathy with left ventricular hypertrabeculation. *Circ. Cardiovasc. Genet.* **2**, 442–449 (2009).

96. H. Kassem, R. S. Azer, M. Saber-Ayad, S. Moharem-Elgamal, G. Magdy, A. Elguindy, F. Cecchi, I. Olivotto, M. H. Yacoub, Early results of sarcomeric gene screening from the Egyptian National BA-HCM Program. *J. Cardiovasc. Transl. Res.* **6**, 65–80 (2013).

97. A. Woo, H. Rakowski, J. C. Liew, M. S. Zhao, C. C. Liew, T. G. Parker, M. Zeller, E. D. Wigle, M. J. Sole, Mutations of the beta myosin heavy chain gene in hypertrophic cardiomyopathy: Critical functional sites determine prognosis. *Heart* **89**, 1179–1185 (2003).

98. S. L. Van Driest, M. A. Jaeger, S. R. Ommen, M. L. Will, B. J. Gersh, A. J. Tajik, M. J. Ackerman, Comprehensive analysis of the beta-myosin heavy chain gene in 389 unrelated patients with hypertrophic cardiomyopathy. *J. Am. Coll. Cardiol.* **44**, 602–610 (2004).

99. S. Greber-Platzer, M. Marx, C. Fleischmann, C. Suppan, M. Dobner, M. Wimmer, Beta-myosin heavy chain gene mutations and hypertrophic cardiomyopathy in Austrian children. *J. Mol. Cell. Cardiol.* **33**, 141–148 (2001).

100. J. Erdmann, S. Daehmlow, S. Wischke, M. Senyuva, U. Werner, J. Raible, N. Tanis, S. Dyachenko, M. Hummel, R. Hetzer, V. Regitz-Zagrosek, Mutation spectrum in a large cohort of unrelated consecutive patients with hypertrophic cardiomyopathy. *Clin. Genet.* **64**, 339–349 (2003).

101. A. A. Alfares, M. A. Kelly, G. McDermott, B. H. Funke, M. S. Lebo, S. B. Baxter, J. Shen, H. M. McLaughlin, E. H. Clark, L. J. Babb, S. W. Cox, S. R. DePalma, C. Y. Ho, J. G. Seidman, C. E. Seidman, H. L. Rehm, CORRIGENDUM: Results of clinical genetic testing of 2,912 probands with hypertrophic cardiomyopathy: Expanded panels offer limited additional sensitivity. *Genet. Med.* **17**, 319 (2015).

102. S. Cuenca, M. J. Ruiz-Cano, J. R. Gimeno-Blanes, A. Jurado, C. Salas, I. Gomez-Diaz, L. Padron-Barthe, J. J. Grillo, C. Vilches, J. Segovia, D. Pascual-Figal, E. Lara-Pezzi, L. Monserrat, L. Alonso-Pulpon, P. Garcia-Pavia, Inherited Cardiac Diseases Program of the Spanish Cardiovascular Research Network (Red Investigación Cardiovascular), Genetic basis of familial dilated cardiomyopathy patients undergoing heart transplantation. *J. Heart Lung Transplant.* **35**, 625–635 (2016).

103. J. I. van Waning, K. Caliskan, Y. M. Hoedemaekers, K. Y. van Spaendonck-Zwarts, A. F. Baas, S. M. Boekholdt, J. P. van Melle, A. J. Teske, F. W. Asselbergs, A. P. C. M. Backx, G. J. du Marchie Sarvaas, M. Dalinghaus, J. M. P. J. Breur, M. P. M. Linschoten, L. A. Verlooij, I. Kardys, D. Dooijes, R. H. Lekanne Deprez, A. S. IJpma, M. P. van den Berg, R. M. W. Hofstra, M. A. van Slegtenhorst, J. D. H. Jongbloed, D. Majoor-Krakauer, Genetics, clinical features, and long-term outcome of noncompaction cardiomyopathy. *J. Am. Coll. Cardiol.* **71**, 711–722 (2018).

104. H. Watkins, L. Thierfelder, D. S. Hwang, W. McKenna, J. G. Seidman, C. E. Seidman, Sporadic hypertrophic cardiomyopathy due to de novo myosin mutations. *J. Clin. Invest.* **90**, 1666–1671 (1992).

105. M. Enjuto, A. Francino, F. Navarro-Lopez, D. Viles, J. C. Pare, A. M. Ballesta, Malignant hypertrophic cardiomyopathy caused by the Arg723Gly mutation in β-myosin heavy chain gene. *J. Mol. Cell. Cardiol.* **32**, 2307–2313 (2000).

106. P. Richard, P. Charron, L. Carrier, C. Ledeuil, T. Cheav, C. Pichereau, A. Benaiche, R. Isnard, O. Dubourg, M. Burban, J.-P. Gueffet, A. Millaire, M. Desnos, K. Schwartz, B. Hainque, M. Komajda, EUROGENE Heart Failure Project, Hypertrophic cardiomyopathy. *Circulation* **107**, 2227–2232 (2003).

107. S. Waldmüller, J. Erdmann, P. Binner, G. Gelbrich, S. Pankuweit, C. Geier, B. Timmermann, J. Haremza, A. Perrot, S. Scheer, R. Wachter, N. Schulze-Waltrup, A. Dermintzoglou, J. Schönberger, W. Zeh, B. Jurmann, T. Brodherr, J. Börgel, M. Farr, H. Milting, W. Blankenfeldt, R. Reinhardt, C. Özcelik, K.-J. Osterziel, M. Loeffler, B. Maisch, V. Regitz-Zagrosek, H. Schunkert, T. Scheffold, German Competence Network Heart Failure, Novel correlations between the genotype and the phenotype of hypertrophic and dilated cardiomyopathy: Results from the German Competence Network Heart Failure. *Eur. J. Heart Fail.* **13**, 1185–1192 (2011).

108. D. M. Seleznev, S. A. Gabrusenko, E. V. Parfenova, V. G. Naumov, D. V. Stambol'skiĭ, V. A. Tkachuk, The role of mutation in cardiac beta-myosin heavy chain gene in population of patients. *Kardiologiia* **45**, 15–20 (2005).

109. J. P. Kaski, P. Syrris, M. T. Esteban, S. Jenkins, A. Pantazis, J. E. Deanfield, W. J. McKenna, P. M. Elliott, Prevalence of sarcomere protein gene mutations in preadolescent children with hypertrophic cardiomyopathy. *Circ. Cardiovasc. Genet.* **2**, 436–441 (2009).

110. K. Poetter, H. Jiang, S. Hassanzadeh, S. R. Master, A. Chang, M. C. Dalakas, I. Rayment, J. R. Sellers, L. Fananapazir, N. D. Epstein, Mutations in either the essential or regulatory light chains of myosin are associated with a rare myopathy in human heart and skeletal muscle. *Nat. Genet.* **13**, 63–69 (1996).

111. J. Flavigny, P. Richard, R. Isnard, L. Carrier, P. Charron, G. Bonne, J. F. Forissier, M. Desnos, O. Dubourg, M. Komajda, K. Schwartz, B. Hainque, Identification of two novel mutations in the ventricular regulatory myosin light chain gene (MYL2) associated with

familial and classical forms of hypertrophic cardiomyopathy. *J. Mol. Med.* **76**, 208–214 (1998).

112. R. E. Hershberger, N. Norton, A. Morales, D. Li, J. D. Siegfried, J. Gonzalez-Quintana, Coding sequence rare variants identified in MYBPC3, MYH6, TPM1, TNNC1, and TNNI3 from 312 patients with familial or idiopathic dilated cardiomyopathy. *Circ. Cardiovasc. Genet.* **3**, 155–161 (2010).

113. T. M. Olson, N. Y. Kishimoto, F. G. Whitby, V. V. Michels, Mutations that alter the surface charge of alpha-tropomyosin are associated with dilated cardiomyopathy. *J. Mol. Cell. Cardiol.* **33**, 723–732 (2001).

114. T. J. Pugh, M. A. Kelly, S. Gowrisankar, E. Hynes, M. A. Seidman, S. M. Baxter, M. Bowser, B. Harrison, D. Aaron, L. M. Mahanta, N. K. Lakdawala, G. McDermott, E. T. White, H. L. Rehm, M. Lebo, B. H. Funke, The landscape of genetic variation in dilated cardiomyopathy as surveyed by clinical DNA sequencing. *Genet. Med.* **16**, 601–608 (2014).

115. G. Frisso, G. Limongelli, G. Pacileo, A. Del Giudice, L. Forgione, P. Calabrò, M. Iacomino, N. Detta, L. M. Di Fonzo, V. Maddaloni, R. Calabrò, F. Salvatore, A child cohort study from southern Italy enlarges the genetic spectrum of hypertrophic cardiomyopathy. *Clin. Genet.* **76**, 91–101 (2009).

116. A. M. Matyushenko, V. V. Nefedova, A. M. Kochurova, G. V. Kopylova, N. A. Koubassova, A. G. Shestak, D. S. Yampolskaya, D. V. Shchepkin, S. Y. Kleymenov, N. S. Ryabkova, I. A. Katrukha, S. Y. Bershitsky, E. V. Zaklyazminskaya, A. K. Tsaturyan, D. I. Levitsky, Novel mutation Glu98Lys in cardiac tropomyosin alters its structure and impairs myocardial relaxation. *Int. J. Mol. Sci.* **24**, 12359 (2023).

117. Y. Man, C. Yi, M. Fan, T. Yang, P. Liu, S. Liu, G. Wang, Identification of a novel missense mutation in the TPM1 gene via exome sequencing in a Chinese family with dilated cardiomyopathy: A case report and literature review. *Medicine* **101**, e28551 (2022).

118. J. England, J. Granados-Riveron, L. Polo-Parada, D. Kuriakose, C. Moore, J. D. Brook, C. S. Rutland, K. Setchfield, C. Gell, T. K. Ghosh, F. Bu'Lock, C. Thornborough, E. Ehler, S. Loughna, Tropomyosin 1: Multiple roles in the developing heart and in the formation of congenital heart defects. *J. Mol. Cell. Cardiol.* **106**, 1–13 (2017).

119. L. Thierfelder, H. Watkins, C. MacRae, R. Lamas, W. McKenna, H. P. Vosberg, J. G. Seidman, C. E. Seidman, α-tropomyosin and cardiac troponin T mutations cause familial hypertrophic cardiomyopathy: A disease of the sarcomere. *Cell* **77**, 701–712 (1994).

120. V. Regitz-Zagrosek, J. Erdmann, E. Wellnhofer, J. Raible, E. Fleck, Novel mutation in the alpha-tropomyosin gene and transition from hypertrophic to hypocontractile dilated cardiomyopathy. *Circulation* **102**, E112–E116 (2000).

121. S. Carballo, P. Robinson, R. Otway, D. Fatkin, J. D. H. Jongbloed, N. de Jonge, E. Blair, J. P. van Tintelen, C. Redwood, H. Watkins, Identification and functional characterization of cardiac troponin I as a novel disease gene in autosomal dominant dilated cardiomyopathy. *Circ. Res.* **105**, 375–382 (2009).

122. R. Hassoun, H. Budde, H. G. Mannherz, M. Lódi, S. Fujita-Becker, K. T. Laser, A. Gartner, K. Klingel, D. Möhner, R. Stehle, I. Sultana, T. Schaaf, M. Majchrzak, V. Krause, C. Herrmann, M. M. Nowaczyk, A. Mügge, G. Pfitzer, R. R. Schröder, N. Hamdani, H. Milting, K. Jaquet, D. Cimiotti, De novo missense mutations in *TNNC1* and *TNNI3* causing severe infantile cardiomyopathy affect myofilament structure and function and are modulated by troponin targeting agents. *Int. J. Mol. Sci.* **22**, 9625 (2021).

123. J. F. Forissier, L. Carrier, H. Farza, G. Bonne, J. Bercovici, P. Richard, B. Hainque, P. J. Townsend, M. H. Yacoub, S. Fauré, O. Dubourg, A. Millaire, A. A. Hagège, M. Desnos, M. Komajda, K. Schwartz, Codon 102 of the cardiac troponin T gene is a putative hot spot for mutations in familial hypertrophic cardiomyopathy. *Circulation* **94**, 3069–3073 (1996).

124. Y. Koga, H. Toshima, A. Kimura, H. Harada, T. Koyanagi, H. Nishi, M. Nakata, T. Imaizumi, Clinical manifestations of hypertrophiccardiomyopathy with mutations in the

cardiac beta-myosin heavy chain gene or cardiac troponin T gene. *J. Card. Fail.* **2**, S97–S103 (1996).

125. L. G. D'Cruz, C. Baboonian, H. E. Phillimore, R. Taylor, P. M. Elliott, A. Varnava, F. Davison, W. J. McKenna, N. D. Carter, Cytosine methylation confers instability on the cardiac troponin T gene in hypertrophic cardiomyopathy. *J. Med. Genet.* **37**, E18 (2000).

126. J. Mogensen, R. T. Murphy, T. Shaw, A. Bahl, C. Redwood, H. Watkins, M. Burke, P. M. Elliott, W. J. McKenna, Severe disease expression of cardiac troponin C and T mutations in patients with idiopathic dilated cardiomyopathy. *J. Am. Coll. Cardiol.* **44**, 2033–2040 (2004).

127. R. E. Hershberger, J. R. Pinto, S. B. Parks, J. D. Kushner, D. Li, S. Ludwigsen, J. Cowan, A. Morales, M. S. Parvatiyar, J. D. Potter, Clinical and functional characterization of TNNT2 mutations identified in patients with dilated cardiomyopathy. *Circ. Cardiovasc. Genet.* **2**, 306–313 (2009).
